# Robust and Tailored 1D/3D Heterojunction for Efficient and Stable Perovskite Solar Cells

**DOI:** 10.1002/advs.202524311

**Published:** 2026-03-10

**Authors:** Wending Hao, Tao Liu, Xu Wang, Luan Li, Ziyu Zhang, Fengqi Sun, Qiang Luo, Ning Wang

**Affiliations:** ^1^ School of Marine Sciences (State Key Laboratory of Marine Resources Utilization in South China Sea) Hainan University Haikou P. R. China

**Keywords:** fluorinated ammonium salt, interfacial charge transfer, perovskite solar cells, robust 1D/3D heterojunction, stability

## Abstract

Operational stability remains a critical challenge for organic–inorganic hybrid perovskite solar cells (PSCs). Recently, the use of low‐dimensional perovskite has emerged as a promising route to enhance the device robustness. It is found that the 1D/3D heterojunction has a significant impact on the stability, while the role of spacer cations in the 1D/3D heterojunction regulation is poorly understood. Here, we report the synthesis of two new types of 1D perovskite single crystals, (BZ)_2_Pb_1.5_I_4_, (TFBZ)PbI_3_, using benzamidinium (BZ) and (trifluoromethyl) benzamidinium (TFBZ) as spacer cations. Our analysis reveals that the trifluoromethyl groups in TFBZ contribute to extensive hydrogen‐bond networks and confer a high dipole moment. These features strengthen the interaction between TFBZ and inorganic [PbI_6_]^4−^ skeleton, resulting in high structure stability and orientationally crystallized 1D perovskites. Furthermore, the incorporation of 1D (TFBZ)PbI_3_ forms a robust and high‐quality 1D/3D heterojunction interface, facilitated by stable 1D phase, favorable lattice matching, strong interface binding, and effective defect passivation. Accordingly, the resulting TFBZ‐based 1D/3D hybrid PSCs achieve a power conversion efficiency of 25.54%, while maintaining expectational operational and thermal stability. This work provides a design strategy to control the microstructure of a 1D/3D heterojunction, enabling highly efficient and stable perovskite photovoltaics.

## Graphical abstract

This study successfully established a robust 1D/3D heterojunction, characterized by a stable 1D phase, favorable lattice matching, strong interface binding, and effective defect passivation. The resulting epitaxial perovskite film demonstrates preferential vertical orientation of (100) facets, thereby facilitating carriers’ transport and stress relief. The strategically engineered 1D/3D heterojunction provides a clear pathway toward high‐performance and durable perovskite photovoltaics.

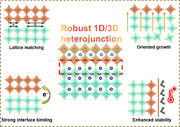

## 1 Introduction

Organic–inorganic hybrid perovskite materials have emerged as promising photovoltaic materials due to their excellent optoelectronic properties [1, 2, 3]. Despite rapid advancements in power conversion efficiencies (PCEs), the commercial viability of perovskite solar cells (PSCs) is hampered by their intrinsic instability under operational stresses such as moisture, heat, oxygen, UV light, and electric field [4, 5, 6, 7]. This instability is rooted in the crystal structure of the 3D polycrystalline perovskites. Their framework consists of corner‐sharing [PbI_6_]^4−^ octahedra, with small interstitial cations (e.g., MA^+^, FA^+^, Cs^+^) stabilized by weak van der Waals interactions. This configuration confers low decomposition activation energy and facilitates ion migration, compromising material stability [8, 9, 10]. Specially, the perovskite lattice readily forms iodine vacancies, which act as channels for accelerated ion diffusion [11]. Unlike the band‐like transport of the high‐mobility electronic charge carriers, the ionic migration in perovskites proceeds through a hopping mechanism, with rates strongly dependent on external stimuli [12, 13]. In this scenario, structure‐induced ion migration is a primary driver of perovskite decomposition and subsequent degradation of device performance under operational environmental restrictions.

Perovskites structures are defined by the diverse arrangements of [PbX_6_]^4−^ octahedra building blocks, which give rise to 0D, 1D, 2D, and 3D perovskite structures [14]. Low‐dimensional (LD) perovskites, which incorporate bulky organic cations, exhibit markedly different optoelectronic characteristics compared to their 3D counterparts, including wider bandgaps and enhanced environmental stability [15, 16]. These attributes have motivated their use as interfacial modifiers in 3D perovskite architectures [17, 18, 19]. In particular, 2D/3D heterostructures, fabricated via additive or interfacial engineering, effectively passivate defects and improve device stability [20]. Nevertheless, 2D perovskites suffer from strong quantum confinement, leading to high exciton binding energies (>300 meV) and poor charge carrier mobility (typically < 10^−^^4^ cm^2^ V^−^^1^ s^−^^1^) [21]. Moreover, horizontally stacked 2D layers can induce charge localization and introduce trap states, particularly in low‐n phases, degrading short‐circuits current density (*J*_sc_) and fill factor (FF) [22]. Furthermore, the insulating organic spacing layers, typically aligned parallel to the substrate, further restrict out‐of‐plane charge transport [23]. Additionally, 2D/3D interfaces are vulnerable to thermal stress, which can drive migration of bulky cations into the 3D lattice, collapsing the heterostructure and degrading device performance [24]. Since 0D perovskites exhibit spatial and electronic discontinuity, charge scattering between isolated [PbX_6_] islands and the 3D host severely restricts carrier transport [25].

By contrast, 1D perovskites featured by 1D inorganic halide octahedra chains provide continuous carrier transport pathways with minimal quantum confinement, yielding carrier mobilities on the order of 10^−2^ cm^2^ V^−1^s^−1^ [26]. Additionally, their low formation energy (ΔE <0.1 eV) effectively suppresses phase transitions and ligand migration, ensuring structural integrity [27]. The diverse octahedral connectivity (corner‐, edge‐, or face‐sharing) and tunable cation steric afford considerable structural flexibility [28]. Hence, these features make 1D perovskites promising stabilizers for PSCs, particularly under thermal stress [29]. Meanwhile, the functional groups on the organic moieties help passivate deep‐level defects and suppress non‐radiative recombination [30]. Gao’ group investigated the formation dynamics of 1D and 2D perovskites derived from the same secondary amine cation of N‐methyl‐1‐(naphthalen‐1‐yl)methylammonium (M‐NMA^+^), revealing that the intermolecular 𝜋–𝜋 stacking of M‐NMA^+^ and their bonding with inorganic PbI_6_ octahedra govern the dimensionality of the resulting phase [31]. Hu and coworkers have introduced ionic liquids on the surface of 3D perovskite to induce 1D perovskite layer, reducing non‐radiative recombination and improving charge transport [32]. Jiu et al. obtained orientationally crystallized 1D perovskite nanorods on FAPbI_3_ via 4‑chlorobenzamidine hydrochloride (CBAH), enhancing thermal, moisture, and illumination stability [33]. LD perovskites or organic cations were also added into perovskite precursors to template the epitaxial growth of 3D perovskites [34]. Good lattice matching between LD and 3D components alleviates relieve residual stress, suppresses defect formation, and improves structural stability [35]. For example, Fan's group incorporated 1D PbI_2_‐BPy into a 3D perovskite precursor to form a lattice‐matched 1D/3D hybrid perovskites, enhancing stability under high electric fields and suppressing ion migration [36]. The well‐designed organic salts by tuning cation size, functional groups, and spatial configuration can precisely tune the crystal structure (e.g., face‐, edge‐, or corner‐sharing connectivity of PbX_6_ octahedra) and microscopic arrangement (e.g., chain spacing tunable and intermolecular distance) of 1D perovskites [37, 38, 39], which hold promise for excellent lattice matching with 3D perovskites. However, the controlled formation of high‐quality 1D/3D heterojunctions remains an open research frontier.

In this study, two kinds of formamidine salt, including benzamidinium hydrochloride (BZCl) and 4‐(trifluoromethyl) benzamidinium hydrochloride (TFBZCl), were reacted with PbI_2_ in hydroiodic acid (HI). This yielded two distinct 1D perovskite single‐crystal products: (BZ)_2_Pb_1.5_I_4_ (n = 2 phase) and (TFBZ)PbI_3_ (n = 1 phase). Furthermore, introducing 1D single crystals into 3D perovskite precursor enable us to investigate the evolution of 1D/3D heterojunction structures. Compared with (BZ)_2_Pb_1.5_I_4_, (TFBZ)PbI_3_ exhibits more pronounced out‐of‐plane orientation in the resulting 1D/3D perovskite films, which may be due to the structural stability of 1D perovskites with an extensive hydrogen‐bond network. Moreover, 1D (TFBZ)PbI_3_ formed a better lattice‐matching 1D/3D heterojunction with the 3D perovskite host than (BZ)_2_Pb_1.5_I_4_. The resulting epitaxial growth enhanced film crystallinity and grain orientation, favoring vertical carrier's transport and stress relief. The organic moieties of 1D (TFBZ)PbI_3_ also contributed to the passivation of deep‐level defects and suppression of ion migration in 3D perovskites. Density functional theory (DFT) calculations revealed a stronger interfacial binding energy in the well‐matched (TFBZ)PbI_3_‐based 1D/3D heterostructure, indicating enhanced charge transfer across the junction. Consequently, PSCs incorporating 1D (TFBZ)PbI_3_ achieved a champion efficiency of 25.54%, attributable to optimized 1D/3D heterojunction interfaces, which allow efficient carrier transport and suppress non‐radiative recombination. These devices also showed superior thermal and operational stability in comparison to both pristine 3D devices and BZ‐based 1D/3D analogues. The current study opens the possibility of controlling the growth of perovskite films by well‐designed 1D/3D heterojunction interface, enabling highly oriented epitaxial growth and robust stability via a low‐cost solution‐based route.

**FIGURE 1:**
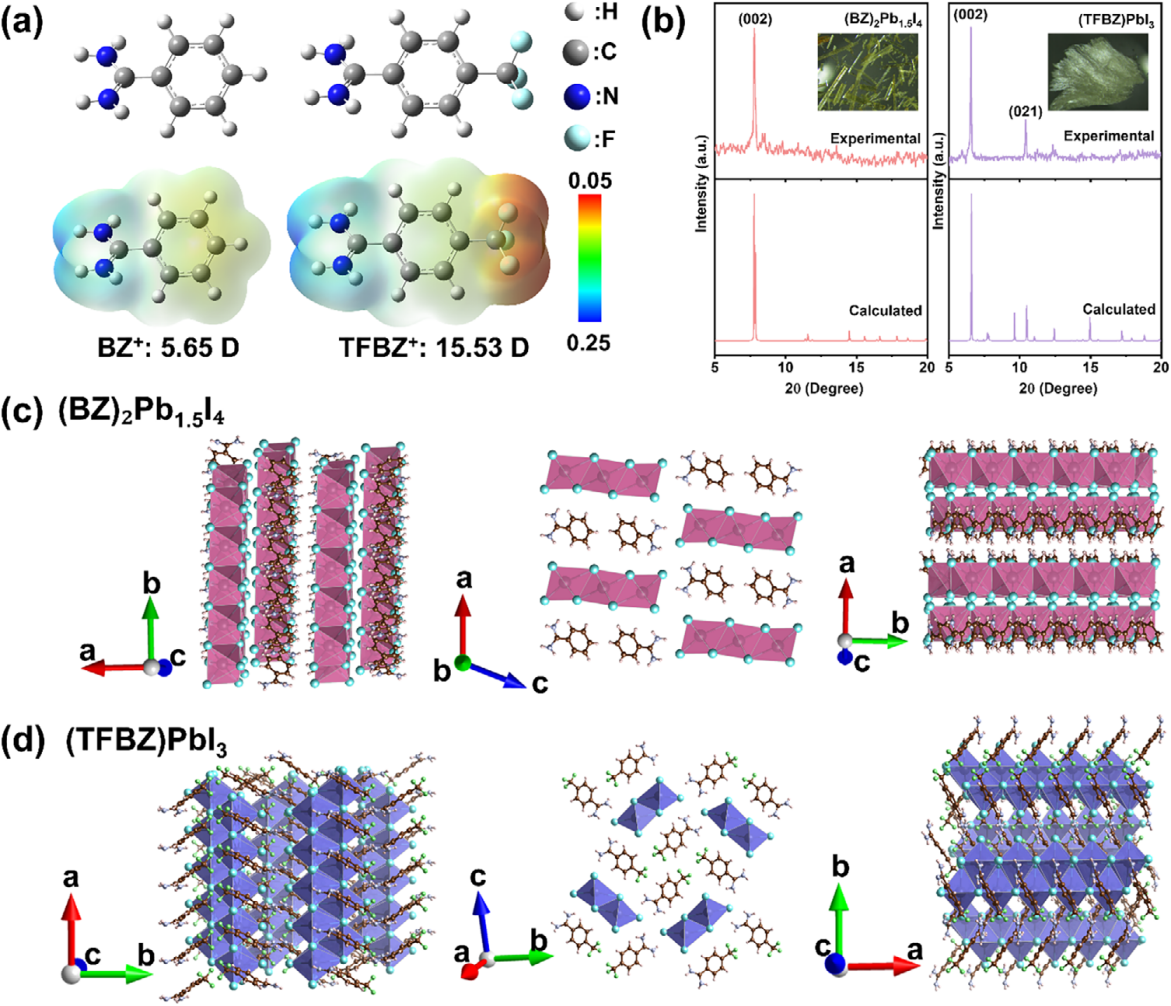
(a) Molecular structures and ESP maps of BZ and TFBZ spacer cations. The dipole moments were calculated using Gaussian software. (b) Experimental and simulated XRD patterns of 1D (BZ)_2_Pb_1.5_I_4_ and (TFBZ)PbI_3_ single crystals. The insets show the corresponding optical microscopy images of 1D (TFBZ)PbI_3_ and (BZ)_2_Pb_1.5_I_4_ single‐crystal powders. Views of the 1D (c) (BZ)_2_Pb_1.5_I_4_ and (d) (TFBZ)PbI_3_ crystal structure along different crystallographic axes.

Herein, we designed and self‐prepared two 1D perovskite single crystals, (BZ)_2_Pb_1.5_I_4_ and (TFBZ)PbI_3_, and incorporated into perovskite precursors to fabricate 1D/3D perovskite films. The detailed experiments can be found in the Experimental Section. DFT calculations reveal that the trifluoromethyl (‐CF_3_) groups in the TFBZ spacer cation act as strong electron‐withdrawing units, resulting in a higher molecular dipole moment (15.53 Debye) than that of BZ (Figure 1a). This enhanced polarity strengthens the interaction between the TFBZ cation and the inorganic [PbI_6_]^4−^ framework, thereby improving structural stability. Compared to BZ, the lowered highest occupied molecular orbital (HOMO) of TFBZ facilitates the extraction and collection of holes (Figure S1). X‐ray diffraction (XRD) patterns and corresponding crystallographic data of 1D perovskites are shown in Figure 1b and Tables S1–S4. The experimental XRD patterns confirm the successful synthesis of both light yellow rod‐shaped 1D crystals, and closely match the calculated patterns derived from single‐crystal data. 1D (TFBZ)PbI_3_ perovskite exhibited two sharp diffraction peaks at 6.58° and 10.42°, assigned to the (002) and (021) planes, respectively. Only one sharp diffraction peak at 7.80° was observed for 1D (BZ)_2_Pb_1.5_I_4_ perovskite, attributed to (002) plane. Rietveld refinement of single‐crystal data shows that (BZ)_2_Pb_1.5_I_4_ crystallizes in a monoclinic C2/c space group, whereas (TFBZ)PbI_3_ adopts a P2_1_/c space group. Both structures feature edge‐sharing [PbI_6_]^4−^ octahedral chains (Figure 1c,d). Structural analysis indicates that the ─CF_3_ group in TFBZ promotes a shorter average Pb–I bond length (3.20 Å vs 3.32 Å in BZ) and a reduced interlayer spacing (4.54 Å vs 4.56 Å), suggesting enhanced lattice rigidity (Figures S2, S3). The electron‐withdrawing ─CF_3_ group also induces a more positive charge on the formamidine moiety of TFBZ, strengthening the NH···I hydrogen bonding with the inorganic framework. This is supported by ^1^H nuclear magnetic resonance, which shows a pronounced downfield shift of the N–H proton resonance in the TFBZ–PbI_2_ system compared to BZ–PbI_2_ and FAI–PbI_2_ (Figure S5). Consistent with this, the NH···I hydrogen bond length is shorter in (TFBZ)PbI_3_ (3.65 Å) than in (BZ)_2_Pb_1.5_I_4_ (3.75 Å) (Figures S4, S5). Additionally, both NH···F and C─H···F─C hydrogen‐bonding interactions enable head‐to‐tail linkage of four TFBZ spacer cations, forming an extensive hydrogen‐bonding network that enhances structural stability and charge transport. In contrast, the weaker NH···I bonding and reliance on intermolecular π–π stacking in fluorine‐free (BZ)_2_Pb_1.5_I_4_ result in inferior stability, Figure S5.

**FIGURE 2:**
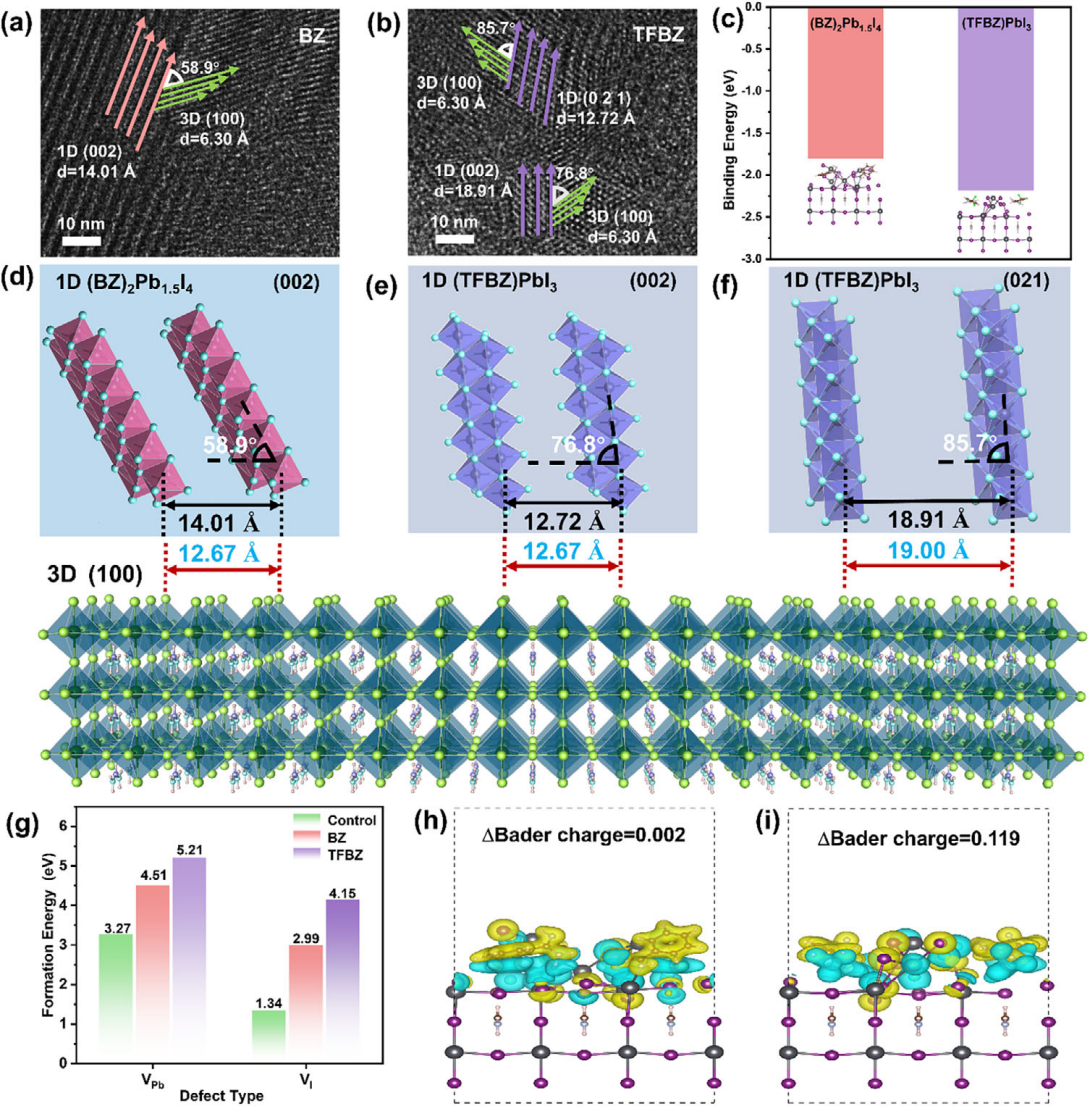
HRTEM images of the heteroepitaxial region for (a) 1D (BZ)_2_Pb_1.5_I_4_/3D and (b) 1D (TFBZ)PbI_3_/3D hybrid perovskites. (c) Binding energies of 1D (BZ)_2_Pb_1.5_I_4_ and (TFBZ)PbI_3_ anchored on the surface of 3D perovskite films. Schematic view of the microstructure of 1D/3D perovskite heterostructure interface with different exposed 1D crystal planes, (d) (002) (BZ)_2_Pb_1.5_I_4_, (e) (002) (TFBZ)PbI_3_, (f) (021) (TFBZ)PbI_3_. (g) Calculated defect formation energies for Pb vacancy (V_Pb_) and I vacancy (V_I_) at the Pb‐I terminated surfaces for the control film, as well as at the 1D (BZ)_2_Pb_1.5_I_4_/3D and 1D (TFBZ)PbI_3_/3D perovskite heterostructure interfaces. Charge density difference plots and Bader charge analysis (yellow: electron accumulation; green: depletion) for 1D (h) (BZ)_2_Pb_1.5_I_4_ and (i) (TFBZ)PbI_3_ on the surface of 3D perovskite films.

High‐resolution transmission electron microscopy (HRTEM) reveals the heterogeneous structure and microscopic morphology of the hybrid 1D/3D perovskite films (Figure 2a,b). For simplicity, films incorporating 1D (BZ)_2_Pb_1.5_I_4_ or (TFBZ)PbI_3_ are termed BZ‐based and TFBZ‐based 1D/3D perovskites, respectively. Macroscopically, 1D and 3D halide perovskites exhibit distinct textures along their preferential orientations, respectively. The interplanar spacing of the (002) plane in 1D (BZ)_2_Pb_1.5_I_4_ measures 14.01 Å, forming a 58.9° angle with the epitaxially grown 3D perovskite (100) plane (Figure 2a). In the TFBZ‐based film (Figure 2b), the (002) and (021) planes of the 1D perovskite show spacings of 18.91 and 12.72 Å, with angles of 76.8° and 85.7° relative to the 3D (100) plane, respectively. These crystallographic data align with XRD results. DFT calculations compare the binding energies of these 1D/3D heterojunctions (Figure 2c). The calculated binding energies for the TFBZ‐based and BZ‐based 1D/3D heterojunction are −2.18 and −1.80 eV, respectively. By contrast, the spontaneous and stable formation of 1D/3D heterostructures contributes to the stronger interfacial binding and enhanced charge transfer dynamics [40]. This high‐quality interface and stable 1D perovskite structure limit the penetration of 1D cations into the perovskite lattice, preserving the corner‐sharing octahedral connections within the 3D framework.

Figure 2d–f provides the schematic view of the microstructure of the 1D/3D halide perovskite based on TEM images, illustrating the lattice matching relations between 1D and 3D perovskites. The (100) crystal plane of the 3D perovskite grows epitaxially on the (002) plane of 1D (BZ)_2_Pb_1.5_I_4_ and on the (002)/ (021) planes of 1D (TFBZ)PbI_3_, facilitated by shared [PbI_6_]^4−^ octahedra. To quantify the heterojunction matching degree, we measured the interplane distance (*d*) between adjacent in‐plane Pb‐atom pair along the 1D crystal planes. For the TFBZ‐based film, the *d*‐value of the (002) plane (12.72 Å) closely matches that of the Pb‐atom pair along the 3D (100) plane (12.67 Å), resulting in minimal lattice mismatch in the heterojunction domain (Figure 2e). Similarly, the 1D (021)/3D (100) heterojunction also exhibits excellent lattice matching (Figure 2f). Here, 1D (TFBZ)PbI_3_ acts as a superior template for 3D epitaxial growth, effectively lowering the nucleation barrier and guiding crystallization toward lower defect density and improved stacking orientation. By contrast, the Pb–Pb distance along the (002) plane of 1D (BZ)_2_Pb_1.5_I_4_ is 14.01 Å, substantially larger than that in the 3D (100) plane, indicating relatively poor lattice matching.

Considering the well‐known defect passivation effects of 1D perovskite, we evaluated their ability to suppress the formation of iodine (V_I_) and lead (V_Pb_) vacancies (structural models in Figure S6). The formation energy of V_Pb_ in the control 3D perovskite is 3.27 eV (Figure 2g). This value increases to 4.51 and 5.21 eV upon incorporation of 1D (BZ)_2_Pb_1.5_I_4_ and (TFBZ)PbI_3_, respectively, confirming that both 1D perovskites effectively inhibit Pb defect formation. The superior V_Pb_ suppression by (TFBZ)PbI_3_ is attributed to a polar covalent interaction between the ─CF_3_ group and Pb^2+^ ions [41], as supported by FTIR and ^19^F NMR spectra (Figure S7). Furthermore, the (TFBZ)PbI_3_‐based hybrid also exhibits the highest V_I_ formation energy, likely due to strong NH···I hydrogen bonding interaction [42]. The structural stability of 1D/3D hybrid perovskite films under thermal stress was evaluated by XRD, Figure S8. For the control sample, thermal ageing at 85°C induced significant decomposition after 150 h. The 1D perovskite phase effectively suppressed the degradation of the perovskite into PbI_2_, functioning both as a surface protective layer against moisture, light, and heat and as a passivator for iodide‐related defects at the grain boundaries (GBs). The XRD patterns of the TFBZ‐based 1D/3D perovskite film remained nearly unchanged after thermal ageing. This enhanced stability can be attributed to the robust 1D/3D heterojunction structure, which benefits from stable 1D (TFBZ)PbI_3_ phase, strong interfacial binding, favorable well lattice matching and effective defect passivation. The charge density difference at the 1D/3D interface is visualized in Figure 2h,i. Consistent with the DFT calculations, the TFBZ‐based heterojunction exhibits a higher electron cloud density, indicating enhanced charge transfer and stronger interfacial binding [43]. This observation is quantified by Bader charge analysis. The ∆Bader charge, defined as the net electron transfer across the interface [44], is significantly larger for the (TFBZ)PbI_3_ heterojunction (0.119) than for the (BZ)_2_Pb_1.5_I_4_ structure (0.002), further confirming more electron transfer and therefore a stronger interface interaction in the former.

**FIGURE 3:**
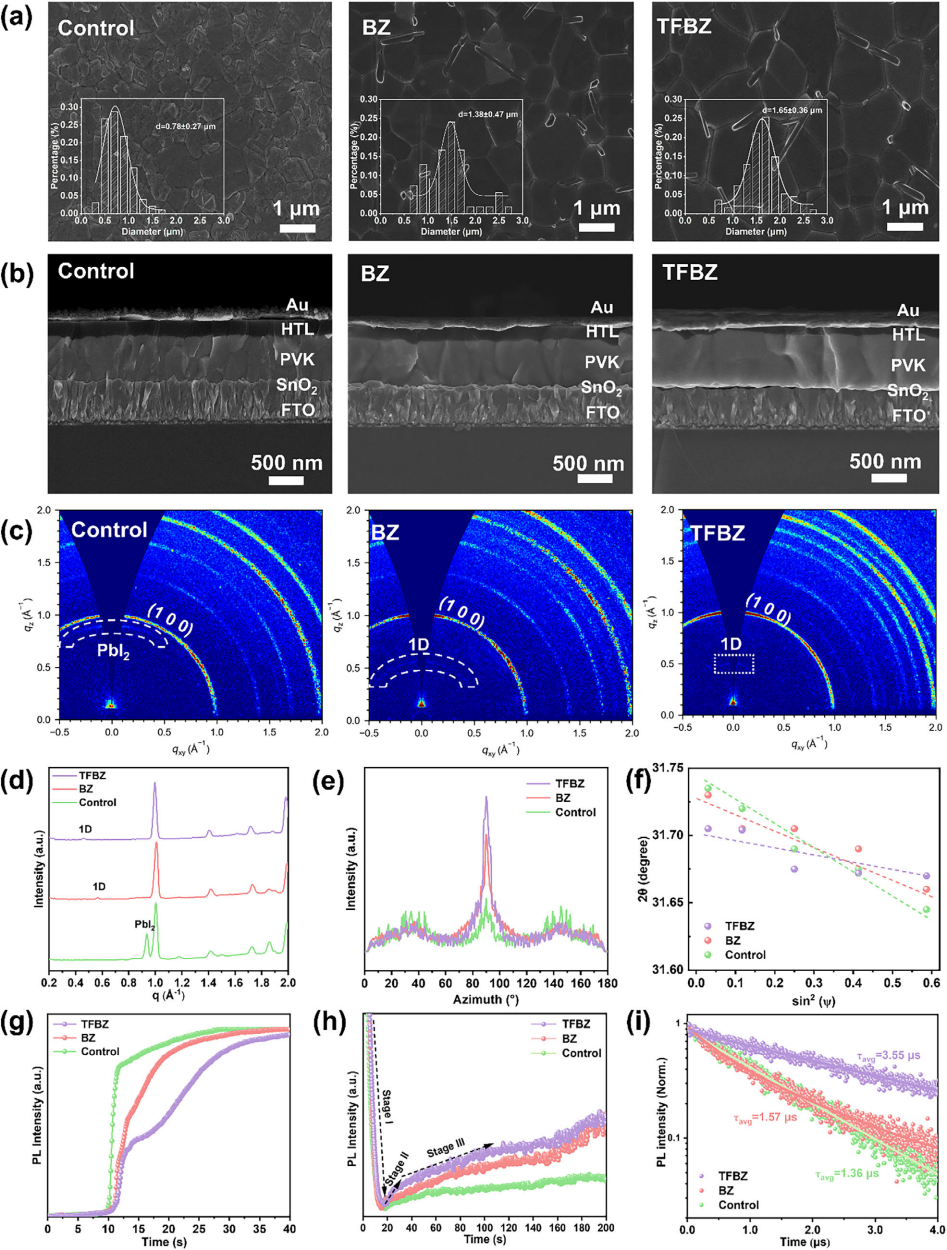
(a) Top‐view and (b) cross‐sectional SEM images of the control, BZ‐treated, and TFBZ‐treated 1D/3D perovskite films. (c) 2D GIWAXS patterns of the control, BZ‐treated, and TFBZ‐treated 1D/3D perovskite films. (d) 1D GIWAXS patterns of the control, BZ‐treated, and TFBZ‐treated perovskite thin films. (e) Azimuth of GIWAXS plots azimuthally along the ring at *q*_xy_ = 10 nm^−1^. (f) Linear fitting of 2θ‐sin^2^(ψ) patterns of the control, BZ‐treated, and TFBZ‐treated 1D/3D perovskite films. Temporal evolution of maximum PL intensity of 1D/3D perovskite films during (g) spin‐coating and (h) annealing. (i) TRPL carrier decay curves of the control, BZ‐treated, and TFBZ‐treated 1D/3D perovskite films.

XRD analysis of the 1D/3D hybrid perovskite films reveals the dependence of the (100) main peak intensity on the 1D perovskite doping concentration. As shown in Figure S9, the (100) peak intensity initially increases and subsequently decreases as the doped concentration rises. The strong and sharp (100) peak signifies enhanced crystallinity, larger grain size, and reduced defect density, which collectively promote charge transport and suppress non‐radiative recombination. An optimal doping concentration of 5 mg/ml was identified for both 1D compositions, at which no residual PbI_2_ or δ‐yellow phase impurities were detected. Further increasing the concentration to 20 mg/ml enables 1D (TFBZ)PbI_3_ and (BZ)_2_Pb_1.5_I_4_ phases to become visible (Figure S10), confirming the preservation of the added 1D perovskite structure within the 1D/3D hybrid film. Morphological characterization by scanning electron microscopy (SEM) revealed the formation of distinctive rod‐like structures on the surfaces of the hybrid films (Figure 3a), indicative of 1D perovskite nanorods. These nanorods were preferentially located at the upper surface and along GBs, which may facilitate vertical charge transport [45]. Both BZ and TFBZ‐doped films exhibited markedly enlarged grain sizes compared to the control, with the (TFBZ)PbI_3_‐treated film achieving the largest average grain size of 1.61 µm. This grain growth is likely attributable to a combination of favorable lattice matching and modified nucleation kinetics. Atomic force microscopy (AFM) further confirmed that incorporation of the 1D perovskites reduced surface roughness, yielding root‐mean‐square (RMS) values of 31.6 nm for BZ‐based film and 22.4 nm for TFBZ‐based film, compared to 59.0 nm for the control film (Figure S11). This smoothing effect is attributed to the compensation provided by the formed 1D perovskite at defect sites [46], which improves the interfacial contact with the hole‐transport layer (HTL). Cross‐sectional SEM images corroborated a significant reduction of GBs domains in the hybrid films (Figure 3b), underscoring effective defect suppression at GBs. Notably, the TFBZ‐based film displayed monolithic grains spanning the entire active layer, which is conducive to efficient vertical carrier extraction and transport.

To probe the influence of 1D perovskite incorporation on film crystallography, we performed 2D grazing‐incidence wide‐angle X‐ray scattering (GIWAXS). The 2D GIWAXS patterns (Figure 3c) and corresponding integrated intensity profiles (Figure 3d) show that scattering peaks at *q* = 9.0 and 10.0 nm^−1^ for the control film correspond to PbI_2_ and 3D perovskite (𝛼‐phase), respectively. By contrast, 1D perovskite treatment results in the disappearance of PbI_2_ and increased 3D perovskite peak intensity, in accordance with our XRD data (Figure S9). New scattering signals emerged at *q* = 5.3 and 6.4 nm^−1^ confirm the presence of (BZ)_2_Pb_1.5_I_4_ and (TFBZ)PbI_3_ 1D phases, respectively. Notably, the (TFBZ)PbI_3_ signal exhibits a pronounced out‐of‐plane orientation along the *q*_z_ direction (Figure 3c), suggesting a preferential vertical self‐assembly that provides a continuous pathway for charge transport [47]. Furthermore, we integrated the GIWAXS pattern azimuthally over the diffraction ring at *q*_xy_ = 1.0 Å^−1^ (Figure 3e). The control film shows three broad peaks at ∼35°, ∼90°, and ∼145°. After 1D perovskite treatment, the peak at ∼90° intensifies and narrows, while the others become weak, indicating preferential vertical stacking of the (100) crystal facets, thereby facilitating carrier transport perpendicular to the substrate [48]. We further quantified residual film stress using depth‐resolved grazing‐incidence X‐ray diffraction (GIXRD). The (012) diffraction peak shifts to lower angles with increasing ψ tilt (Figure S12), indicating tensile stress within all films [49]. This peak shift is less pronounced in 1D/3D hybrid films, with the TFBZ‐based sample showing the smallest variation. Linear fitting of the 2θ–sin^2^(ψ) data (Figure 3f) yields a residual tensile stress of only 37.20 MPa for the TFBZ‐based film, substantially lower than that of the BZ‐based (52.76 MPa) and control (83.90 MPa) films. The higher stress in the latter films implies greater lattice distortion and defect density [50]. The minimal stress in the TFBZ‐based film stems from a well‐lattice‐matched 1D/3D heterojunction, which is proven to be beneficial for defect reduction, as our previous findings.

Dynamic light scattering (DLS) of the 1D/3D perovskite precursor solutions revealed colloidal aggregate exceeding 1 µm in size (Figure S13), contrasting with the sub‐10 nm particles in the control solution. This confirms the persistence of colloidal 1D single crystals in solution. Photoluminescence (PL) spectroscopy further revealed a weak emission signature of the 1D phase, detected exclusively from the film's front surface (Figure S14). This spatial localization indicates that 1D perovskite seeds accumulate at the upper interface and act as an epitaxial template for the downward crystallization of the 3D perovskites [51]. In situ PL spectroscopy was performed for real‐time monitoring of the crystallization dynamics process of perovskite films during spin coating and subsequent thermal annealing (Figure S15). The corresponding PL peak positions and intensities were extracted and plotted in Figure 3g,h. Upon anti‐solvent dripping at ∼10 s, all samples exhibited a rapid rise in PL intensity, signifying α‐phase crystallization. The TFBZ‐based film, however, displayed the lowest initial intensity with a continuous increase, suggesting that TFBZ slows nucleation and crystal growth during spin‐coating [52]. We attribute this moderation to dipole interactions between TFBZ and the [PbI_6_]^4−^ octahedral framework, which can be supported by X‐ray photoelectron spectroscopy (XPS) (Figure S16) and ^1^H NMR (Figure S5) data. During thermal annealing, all films showed an initial PL intensity drop (Stage I), consistent with solvent escape and surface dissolution. In the subsequent recrystallization phase (Stage II), the 1D/3D hybrid films not only recovered more intensity than the control but also exhibited a steadier increase, with the TFBZ‐based film ultimately achieving the highest final PL intensity (Stage III). This enhanced crystallization trajectory is attributed to improved lattice matching, effective defect passivation, and the stable structure of the 1D perovskite. We performed time‐resolved photoluminescence (TRPL) to probe the radiative recombination and carrier transfer behaviors within perovskite films. TRPL decay curves of perovskite films were fitted with a biexponential function (Figure 3i). As listed in Table S5, The TFBZ‐based film shows a substantially longer average carrier lifetime (*τ*_avg_) of 3.55 µs than the control (1.36 µs) and BZ‐treated films (1.57 µs). This directly corroborates the superior suppression of non‐radiative recombination afforded by the (TFBZ)PbI_3_ modification [53].

**FIGURE 4:**
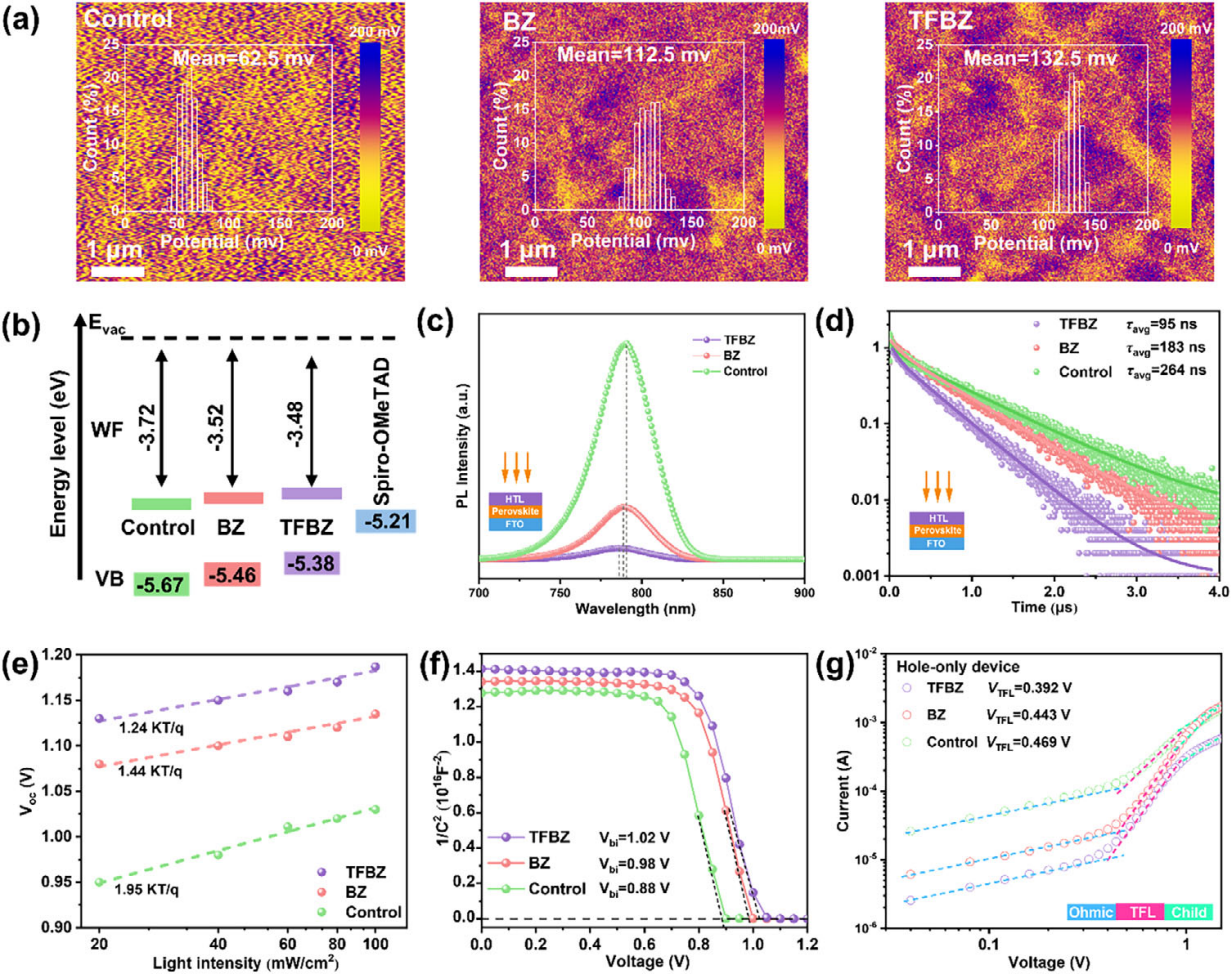
(a) KPFM surface potential maps of the control, BZ‐treated, and TFBZ‐treated perovskite films. (b) Schematic of the energy band alignment. (c) Steady‐state and (d) TRPL spectra of the FTO/PVK/HTL samples. (e) *V*_oc_ versus light intensity plots for the control, BZ‐based and TFBZ‐based devices. (f) Mott–Schottky plots of the control, BZ‐treated, and TFBZ‐treated devices. (g) Dark *I*–*V* characteristics of the hole‐only devices consisting of FTO/PTAA/Perovskite/Spiro‐OMeTAD/Ag.

To elucidate the influence of the 1D perovskite on the electrical properties of the 1D/3D hybrid perovskite films, we performed Kelvin probe force microscopy (KPFM). The TFBZ‐based film exhibited the highest surface potential (132.5 mV), followed by the BZ‐based (112.5 mV) and control (62.5 mV) films (Figure 4a), indicating that the work function (W_F_) of the perovskite's surface was raised by 1D (TFBZ)PbI_3_, which is expected to facilitate hole extraction and transfer in completed devices. The energy‐level alignment was further quantified using ultraviolet photoelectron spectroscopy (UPS) and UV–vis spectroscopy (Figures S17, S18). Consistent with the KPFM results, the W_F_ value increased from −3.72 eV for the control to −3.52 eV for the BZ‐based and −3.48 eV for the TFBZ‐based film (Figure 4b). This shift is attributed to charge redistribution induced by epitaxial growth [54]. Concomitantly, the valence band maximum (VBM) shifted upwards from −5.67 eV to −5.46 eV and −5.38 eV, respectively, signaling a movement of the Fermi level (*E*_F_) toward the valence band and an enhanced p‐type character at the surface [55]. This energy‐level evolution promotes the formation of an additional built‐in electric field, which facilitates hole extraction and suppresses electron transfer at the perovskite/HTL interface, thereby mitigating interfacial recombination. To directly probe charge extraction, we conducted steady‐state and time‐resolved photoluminescence (TRPL) measurements on perovskite films coated with the hole‐transport layer (spiro‐OMeTAD). The TFBZ‐based film showed the most significant PL quenching (Figure 4c), implying highly efficient hole injection into the HTL [56]. This enhancement is ascribed to improved band alignment, strong 1D/3D interfacial binding, and the favorable out‐of‐plane orientation of the 1D phase. Accordingly, the TRPL decay of the TFBZ‐based perovskite/HTL stack yielded the shortest average carrier lifetime (*τ*_avg_ = 95.64 ns, Table S6), confirming the most efficient hole extraction and the strongest suppression of non‐radiative recombination at this critical interface (Figure 4d).

The non‐radiative voltage loss at the interface was evaluated by examining the open‐circuit voltage (*V*_oc_) dependence on light intensity (Figure 4e). According to the ideal diode equation [57], *qV_oc_* ═ *E_g_* − *nkT* *ln*(*I*_0_/*I*), the ideality factor (*n*) could be derived from the slop (*nkT*/*q*) of *V*_oc_ light intensity linear plots, where *E*_g_ is the bandgap of perovskite absorber, *k* is the Boltzmann constant, *q* is the unit charge constant, *T* is the temperature, *I* is the light intensity, and *I*_0_ is a constant with the same unit as *I*. The calculated *n* value decreased from 1.95 (control) and 1.44 (BZ‐based) to 1.24 for the TFBZ‐based device. This lower ideality factor indicates a substantial suppression of trap‐assisted recombination at the perovskite/HTL interfacial [58], which we attribute to the effective defect passivation from 1D (TFBZ)PbI_3_ accumulated on the perovskite upper surface. To unravel the origin of this improvement, we performed a suite of electrochemical measurements, including electrochemical impedance spectroscopy (EIS), dark current leakage, and Mott–Schottky analysis. Electrochemical impedance spectroscopy (EIS) revealed a lower charge‐transfer resistance (*R*_ct_) and a higher recombination resistance (*R*_rec_) in the TFBZ‐based device (Figure S19 and Table S7), pointing to facilitated charge extraction and suppressed recombination, attributed to good lattice‐matching, efficient defect passivation, and optimized energy level alignment. This was further corroborated by dark current‐voltage measurements, where the TFBZ device showed the lowest leakage current (Figure S20) onsistent with reduced shunt loss and a higher measured *J*_sc_. Mott–Schottky analysis was employed to quantify the built‐in potential (*V*_bi_) within the devices (Figure 4f). The *V*_bi_ values were acquired by fitting the capacitance–voltage data to the Mott–Schottky equation: *C* ^−2^═  2(*V_bi_*‐*V_a_*)/*A* ^2^ *e*εε_0_ *N_A_*, where *N_A_* and *V_a_* are the carrier concentration and applied voltage, respectively; ε_0_ and ε denote the vacuum and relative permittivity, respectively; and *e* is the elementary charge [59]. The TFBZ‐based device yielded the largest *V*_bi_ (1.02 V), compared to 0.88 V (control) and 0.98 V (BZ‐based). This enhanced field promotes more efficient charge separation and collection, directly contributing to the *V*_oc_ improvement. Finally, space‐charge‐limited current (SCLC) measurements performed on hole‐only devices (FTO/PTAA/perovskite/Spiro‐OMeTAD/Ag) confirmed a progressive reduction in trap density [60], from 6.21 × 10^15^ cm^−3^ (control) to 5.83 × 10^15^ cm^−3^ (BZ) and 5.27 × 10^1^ ^5^ cm^−3^ (TFBZ), as evidenced by the decreasing trap‐filled limit voltage (*V*_TFL_) (Figure 4g). This trend aligns consistently with the enhanced charge‐transfer and extraction properties conferred by the 1D perovskite modification.

**FIGURE 5:**
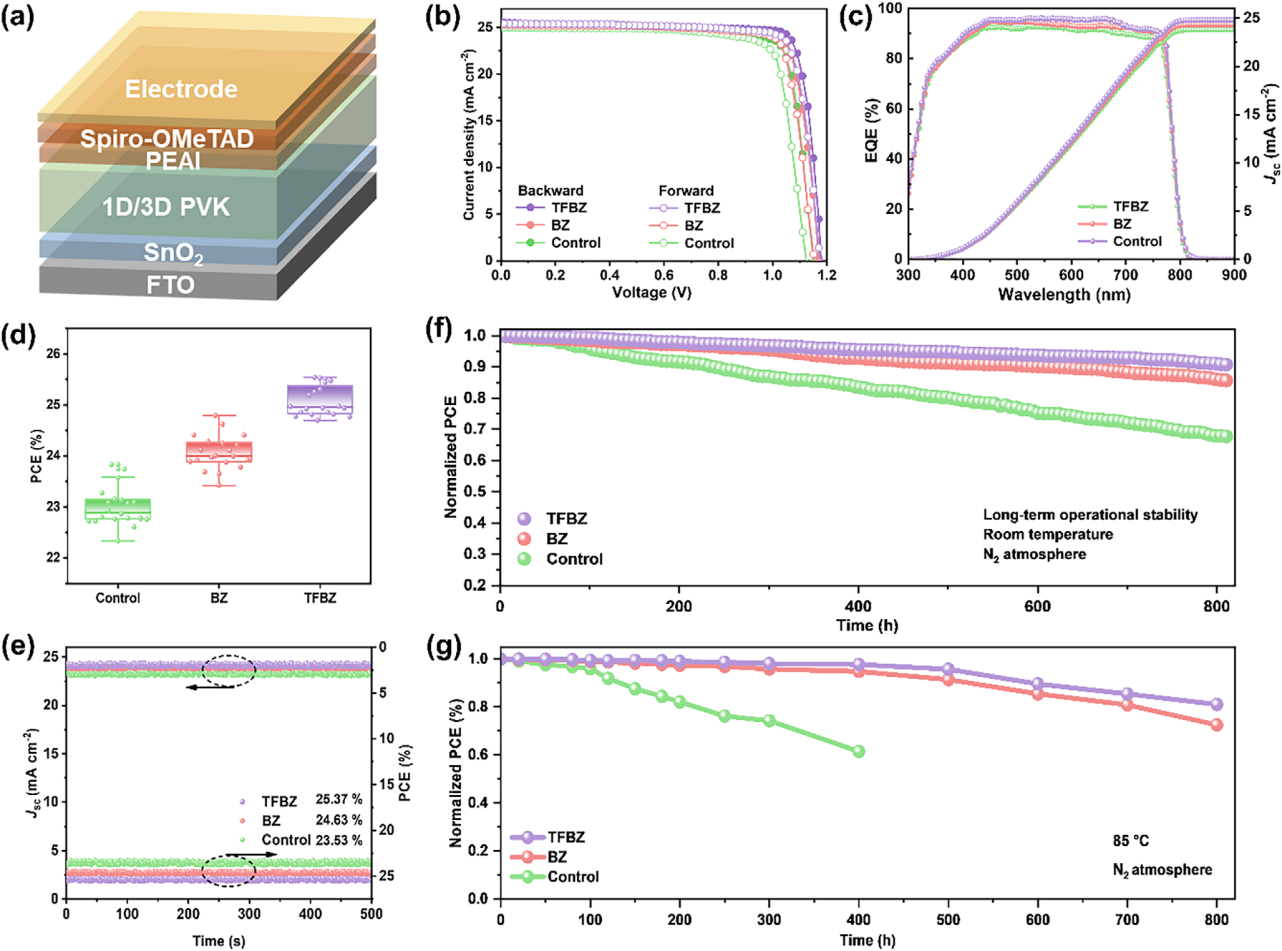
(a) Schematic diagram of the device structure. (b) *J*–*V* characteristics of the champion control, BZ‐based, and TFBZ‐based devices. (c) EQE spectra and integrated *J*_sc_ values. (d) PCE statistics from 20 individual cells. (e) Steady‐state power output of the control, BZ‐based, and TFBZ‐based devices. (f) MPP tracking tests of the unencapsulated devices under 1 sun illumination in N_2_ atmosphere. (g) Long‐term stability of unencapsulated devices heated at 85°C in a N_2_ atmosphere in the dark.

To investigate the effect of 1D perovskite incorporation on the photovoltaic performance of 1D/3D hybrid PSCs, we fabricated n‐i‐p structured PSCs (FTO/SnO_2_/perovskite/PEAI/HTL/Au, Figure 5a). The champion TFBZ‐based device achieved a power conversion efficiency (PCE) of 25.54% (*V*_oc_ ═ 1.18 V, *J*_sc_ ═ 25.47 mA cm^−2^, FF ═ 85%), outperforming the BZ‐based (24.79%) and control (23.80%) devices (Figure 5b; Table S8). This performance enhancement underscores the dual benefit of the stable 1D phase and the improved 1D/3D heterojunction. The integrated current densities derived from external quantum efficiency (EQE) spectra were 24.09, 24.45, and 24.83 mA cm^−2^ for control, BZ‐based, and TFBZ‐based devices, respectively (Figure 5c), matching well with the measured *J*_sc_ from *J–V* measurements. Statistical analysis of twenty independently fabricated devices confirmed excellent reproducibility, with average PCEs increasing from 22.81% (control) to 24.15% (BZ) and 25.22% (TFBZ) (Figure 5d; Figure S21). Furthermore, maximum power point (MPP) tracking yielded stabilized PCE outputs of 25.37%, 24.63%, and 23.53% for the TFBZ‐, BZ‐, and control‐based devices, respectively (Figure 5e). Beyond efficiency, the 1D perovskite markedly enhanced device longevity. Under continuous MPP tracking in a N_2_ atmosphere, the unencapsulated TFBZ‐based device retained 90.74% of its initial PCE after 800 h, a significant improvement over the control and BZ‐based devices (Figure 5f). This operational stability is attributed to the robust 1D/3D heterostructure, which suppresses interfacial recombination and ion migration. This strategy also conferred superior thermal resilience. The spiro‐OMeTAD was replaced by a more thermally stable HTL of poly[bis(4‐phenyl) (2,4,6‐trimethylphenyl) amine] (PTAA) in thermal stability tests. When aged at 85°C in N_2_, the control device retained only 61.47% of its initial PCE after 400 h. However, the TFBZ‐based maintained 81.10% of its initial PCE after 800 h, larger than that of BZ‐based devices (72.57%), Figure 5g. Under ambient conditions with moderated relative humidity (40% RH), the unencapsulated 1D/3D PSCs demonstrated significantly enhanced stability, retaining over 96% of their initial efficiency over 400 h, while the PCE of the control device decayed to 61% of the original value (Figure S22). These findings collectively demonstrate that the robust 1D/3D heterojunction, characterized by stable 1D phase, good lattice matching, strong interface binding and effective defect passivation, is an effective strategy for achieving high‐performance and exceptionally stable perovskite photovoltaics.

## 2 Conclusions

In summary, we engineered two distinct 1D/3D hybrid perovskites by incorporating controlled amounts of 1D (TFBZ)PbI_3_ or (BZ)_2_Pb_1.5_I_4_ into a 3D perovskite precursor. The ─CF_3_ functional groups in TFBZ spacer cations create abundant hydrogen‐bond networks and confer a high molecular dipole moment, strengthening its interaction with the inorganic [PbI_6_]^4−^ skeleton. This interaction not only stabilizes the 1D perovskite but also promotes preferential out‐of‐plane orientation, providing uninterrupted charge transport pathway. Compared to its BZ counterpart, the 1D (TFBZ)PbI_3_ phase establishes a lattice‐matched 1D/3D heterojunction interface, which improves crystallization quality, alleviates residual stress, and effectively reduces defect density. TFBZ‐based 1D/3D perovskite films exhibit a favorable facet orientation and stacking mode that facilitate vertical carrier's transport. Furthermore, the 1D (TFBZ)PbI_3_ component optimizes the energy band alignment and strengthens the 1D/3D interfacial binding, leading to highly efficient hole extraction at the perovskite/HTL junction. Consequently, the resulting TFBZ‐based PSCs with robust 1D/3D heterojunction achieve a synergistic enhancement of both efficiency and operational stability. This work establishes the rational design of 1D perovskite single crystals as a viable strategy for constructing high‐quality 1D/3D heterojunctions, offering a clear route to high‐performance and durable perovskite photovoltaics.

## Conflicts of Interest

The authors declare no conflicts of interest.

## Supporting information




**Supporting File 1**: advs74773‐sup‐0001‐SuppMat.docx.

**Supporting File 2**: advs74773‐sup‐0002‐Data.zip.

## Data Availability

The data that support the findings of this study are available from the corresponding author upon reasonable request.
