# Supplementary material for: Robust and Tailored 1D/3D Heterojunction for Efficient and Stable Perovskite Solar Cells
Source: Adv Sci (Weinh). 2026 Mar 10;13(29):e24311. doi: 10.1002/advs.202524311 (PMC13205900; doi:10.1002/advs.202524311)
Supplement: Supplementary file 1 — Supporting File 1: advs74773‐sup‐0001‐SuppMat.docx. [file ADVS-13-e24311-s002.docx]

**Supporting Information**

**Robust and Tailored 1D/3D Heterojunction for Efficient and Stable Perovskite Solar Cells**

*Wending Hao, Tao Liu*,** Xu Wang*, *Luan Li, Ziyu Zhang, Fengqi Sun, Qiang Luo,** *Ning Wang**

W. Hao, Prof. T. Liu, X. Wang, L. Li, Z. Zhang, F. Sun, Prof. Q. Luo, Prof. N. Wang

School of Marine Sciences (State Key Laboratory of Marine Resources Utilization in South China Sea),

Hainan University,

Haikou 570228, P. R. China

* Corresponding author. E-mail: [liutao565@126.com](mailto:liutao565@126.com); [luo-q11@foxmail.com](mailto:luo-q11@foxmail.com); [wangn02@foxmail.com](mailto:wangn02@foxmail.com)

**Supporting Information**

**Table of Contents**

[**1. Materials 1**](#_Toc208487336)

[**2. Device Fabrication 1**](#_Toc208487337)

[**3. Characterization 3**](#_Toc208487338)

[**4. Calculations 8**](#_Toc208487339)

[**5. Figures 9**](#_Toc208487340)

[**6. Tables 30**](#_Toc208487341)

[**7. References 36**](#_Toc208487342)

# **Materials**

Fluorine-doped Tin Oxide (FTO) glass substrates, lead (II) bromide (PbBr_2_, 99.99%), formamidinium iodide (FAI, ≥99.5%), methylammonium bromide (MABr, ≥99.0%), and methylamine hydrochloride (MACl, ≥98.0%) were purchased from Sigma-Aldrich. Hydroiodic acid (HI, 57 wt.% in H_2_O), Hypophosphorous acid (H_3_PO_2_, 50 wt.% in H2O), Benzenylamidine (BZ, 98%), lead iodide (PbI_2_, 99.99%), and 4-trifluoromethyl benzamidine hydrochloride (TFBZ, 98%) were purchased from Shanghai Aladdin Biochemical Technology Co., Ltd. Lithium bis(trifluoromethanesulfonyl)imide (Li-TFSI, 99%), FK209-Co (III)-TFSI (≥98.0%), Tin (IV) oxide colloid precursor (SnO_2_, 15% in H_2_O colloidal dispersion), Phenethylamine hydroiodide (PEAI), 2,2',7,7'-Tetrakis[N,N-di(4-methoxyphenyl)amino]-9,9'-spirobifluorene (Spiro-OMeTAD, 99.5%), Poly[bis(4-phenyl) (2,4,6-trimethylphenyl) amine] (PTAA) and 4-tert-butylpyridine (tBP, ≥96.0%) were purchased from Xi’an Polymer Light Technology Co., Ltd. Solvents including isopropanol (IPA, anhydrous, 99.5%), N,N-dimethylformamide (DMF, 99.8%), dimethyl sulfoxide (DMSO, ≥99.9%), and chlorobenzene (CB, 99.8%) were also procured from Sigma-Aldrich. All chemicals were used as received without further purification.

# **Device Fabrication**

- 1. **1D perovskite single crystal preparation**

***2.1.1 1D (BZ)_2_Pb_1.5_I_4_ single crystals***

BZ (0.120 g) and PbI_2_ (0.231 g) was mixed with a molar ratio of 2:1 and added in a mixture of hypophosphorous acid (1 mL) and hydroiodic acid (5 mL). The solution was placed in a clear glass vial and maintained at 180 °C for 1 hour to facilitate dissolution and nucleation. Subsequently, the temperature was gradually reduced at a controlled rate of 3 °C/h until room temperature (25 °C) was reached, yielding high-quality (BZ)_2_Pb_1.5_I_4_ single crystals.

***2.1.2 1D (TFBZ)PbI_3_ single crystals***

TFBZ (0.225 g) and PbI₂ (0.231 g) were mixed with a molar ratio of 2:1 and dissolved in a solvent mixture consisting of H_3_PO_2_ (1 mL) and HI (5 mL). The reaction was carried out in a clear glass vial under isothermal conditions at 180 °C for 1 hour. The system was then subjected to a programmed cooling process at a rate of 2 °C/h down to 25 °C, resulting in the formation of 1D (TFBZ)PbI_3_ single crystals with high phase purity and well-defined morphology.

**2.2 Perovskite precursor preparation**

For the (FA_0.95_MA_0.05_Pb(I_0.95_Br_0.05_)_3_) perovskite composition, precursors including PbI_2_ (706.9 mg), PbBr_2_ (27.05 mg), FAI (240.76 mg), MABr (8.21 mg), and MACl (33.76 mg) were dissolved in 1 mL of a mixed solvent of DMF and DMSO (4:1, v/v). The three-dimension (3D) perovskite precursor solution was stirred overnight to ensure complete dissolution of perovskite components. Different numbers of one-dimension (1D) perovskite single crystals were added into 3D perovskite precursor and stirred for 2 h to obtain 1D/3D perovskite precursor. The final precursor solution was filtered through a 0.22 μm polytetrafluoroethylene filter before use.

**2.3 Fabrication of perovskite solar cells**

The FTO glass substrates were initially scrubbed with detergent, followed by sequential sonication in deionized water, acetone, and isopropanol for 15 minutes each. After drying, the substrates underwent a 20-minute UV ozone treatment before further processing. A diluted SnO_2_ aqueous solution (1:3 w/w) was deposited onto the pre-cleaned FTO substrates via spin coating at 4000 rpm for 30 seconds, and subsequently annealed in ambient air at 150 °C for 30 minutes on a hot plate. After cooling to room temperature, the substrates were transferred to a nitrogen-filled glove box. Subsequently, the perovskite precursor was deposited via a two-step spin-coating procedure at 2000 rpm for 10 s, followed by 6000 rpm for 45 s. An antisolvent was dripped onto the spinning substrate 15 s before the end of the second step to facilitate crystallization. The resulting film was annealed at 110 °C for 60 minutes to form a highly crystalline perovskite layer. After cooling to room temperature, a 5 mg/mL isopropanol solution containing PEAI was spin-coated onto the surface of the perovskite film at 5000 rpm for 40 seconds. Thereafter, the Spiro-OMeTAD layer was subsequently deposited on the as prepared perovskite layer by spin-coating of 60 μL of stock solution, which was composed of 90 mg Spiro-OMeTAD in 20 μL Li-TFSI solution (520 mg Li-TFSI in 1 mL ACN), 8 μL FK209- Co (III)-TFSI solution (300 mg in 1 mL ACN), 36 μL tBP and 1 mL CB, at 4000 rpm for 30 s. Finally, Au electrode with a thickness of ca. 80 nm was evaporated on the top of the Spiro-OMeTAD layer using a thermal evaporator.

# Characterization

- 1. **Material characterization**

X-ray diffraction (XRD) measurements were performed on the perovskite films in regular θ−2θ scanning mode by an X-ray diffractometer (Rigaku, D-Max 2200) with a radiation source of Cu Kα1 line (λ = 0.1542 nm). Data were collected in Bragg–Brentano geometry over a 2θ range of 10–80° with a step size of 0.01°. The single-crystal diffraction data are collected by the single-crystal X-ray diffractometer produced by Bruker (D8 Venture). Data integration and structure refinement were performed using the APEX3 software and the SHELX package. The surface morphology of the perovskite films and cross-sectional view of the devices were characterized using a scanning electron microscope (Hitachi, S-4800). High-resolution transmission electron microscopy (HRTEM) was conducted on FEI Talos 200S instrument operating at 200 kV. Samples were prepared by drop-casting dispersions onto ultrathin carbon-coated copper grids. Surface topography and roughness were analyzed by atomic force microscopy (AFM) on a Bruker Dimension Icon system in tapping mode. Scans were performed over 5 × 5 μm² areas with a resolution of 512 × 512 pixels. The surface potential of the perovskite film was acquired by the scanning Kelvin probe force microscopy (SKPM) (Bruker Dimension Icon). The size distribution was acquired by laser particle size analyzer (Zetasizer Nano ZSE). X-ray photoelectron spectroscopy (XPS) and ultraviolet photoelectron spectroscopy (UPS) were measured by Thermo Scientific ESCALAB 250Xi spectrometer using a monochromatized Al Kα radiation source (*hν*=1486.6 eV). The binding energy scale was calibrated by referencing the C 1s peak to 284.8 eV. Fourier-transform infrared (FTIR) spectra were recorded on a Thermo Fisher Nicolet iN10 microscope in transmission mode, with a spectral resolution of 4 cm^-1^ across the range of 4000-400 cm^-1^. Solution-state nuclear magnetic resonance (NMR) spectra were acquired on a Bruker Avance NEO 400 MHz spectrometer using deuterated dimethyl sulfoxide (DMSO‑d₆) as the solvent. The optical absorption spectra of perovskite films were acquired by an ultraviolet/visible/ near-infrared (UV−vis−NIR) spectrophotometer (PerkinElmer, LAMBDA 750). Grazing-incidence wide-angle X-ray scattering (GIWAXS) measurements were performed on a Xeuss 3.0 at a grazing incident angel of 0.2°. Glancing incident angle X-ray diffraction (GIXRD) was performed on a D8-discover 25 diffractometer (Bruker).

**3.2 Photovoltaic performance characterization**

Current density–voltage (*J*–*V*) characteristics were measured under AM 1.5G illumination (100 mW cm⁻²) using a Newport Oriel Sol3A solar simulator, which was calibrated with a certified silicon reference cell. Bias voltages ranging from –0.2 V to 1.2 V were applied using a Keithley 2400 source meter (USA). Incident photon-to-electron conversion efficiency (IPCE) spectra were obtained with a quantum efficiency measurement system (QEX10, PV Measurements). The stabilized power output was evaluated by monitoring the output at a fixed voltage near the maximum power point (MPP), as determined from the *J*–*V* curve. MPP tracking was carried out under 1 sun illumination at 35–40 °C using a white LED solar simulator (Guangzhou Crysco Equipment Co., Ltd.). Electrochemical impedance spectroscopy (EIS) was conducted on a Zahner IM6e electrochemical workstation in the frequency range of 0.1 Hz to 100 kHz, with a DC bias of 0.9 V. Mott–Schottky analysis was performed at 1 kHz under dark conditions. The trap-state density (*N*_t_) was estimated using the space-charge limited current (SCLC) method with hole-only devices having the structure of FTO/SnO₂/Perovskite/PCBM/Ag ^[1]^.

$\text{N}_{\text{trap}}\text{=}\frac{2\text{V}_{\text{TFL}}\varepsilon_{0}\varepsilon_{r}}{eL^{2}}$ (1)

where *V*_TFL_ is the trap-filled-limit voltage, *ε*_0_ is the vacuum permittivity (*ε*_0_ =8.854×10^-14^ F/cm), *e* is the electron charge (*e*=1.602×10^-19^ C) and *L* is the thickness of the perovskite film (*L*≈600 nm). At low light intensities, the open-circuit voltage (*V*_oc_) has a basically linear relationship with the light intensity (*I*_light_). Their approximate relationship can be described by the Shockley equation:

$\text{V}_{\text{oc}}=\frac{kT}{q}\ln\left(\frac{I_{light}}{I_{0}}+1\right)$ (2)

​where *k* represents the Boltzmann constant, *T* denotes the temperature in Kelvin, *q* is the electric charge of an electron, and *I*_0_ is the reverse saturation current. From this expression, it can be seen that the *V*_OC_ increases with the light intensity. However, when the light intensity reaches a certain level, the increment of *V*_OC_ will gradually decrease and tend to saturate. This is because at high light intensities, the trap states are filled, and non-radiative recombination is aggravated, limiting the further increase of the *V*_OC_. Steady-state and time-resolved photoluminescence (PL) measurements were performed on an Edinburgh FLS1000 spectrophotometer equipped with a 450 W xenon lamp and a microsecond flash lamp. Fluorescence lifetimes were determined using time-correlated single-photon counting (TCSPC). The carrier recombination lifetimes can be derived from the TRPL by a bi-exponential fitting shown in Eq. 3^[2]^

$\text{Y}=A_{1}e^{\left(-\frac{t}{\tau_{1}}\right)}+A_{2}e^{\left(-\frac{t}{\tau_{2}}\right)}$ (3)

where $\tau$ is the carrier recombination lifetime, $\tau_{1}$ and $\tau_{2}$ is the decay components of the trap-assisted and radiative recombination process, respectively. The carrier recombination lifetime can be calculated by Eq. 4

$\tau=\frac{A_{1}\tau_{1}^{2}+A_{2}\tau_{2}^{2}}{A_{1}\tau_{1}+A_{2}\tau_{2}}$ (4)

The *in-situ* photoluminescence (PL) measurement system used in this work was a self-constructed setup integrating three core modules: an excitation unit, a fiber-optic light guidance assembly, and a spectrometric detection system. These components operated in coordination to enable high-sensitivity, real-time PL acquisition. A 405 nm laser diode with a maximum output power of 20 W served as the excitation source. The laser beam was focused and directed onto the perovskite sample surface through a customized optical fiber path. The resulting PL emission from the sample was collected via a separate optical fiber and channeled into an Ocean Optics USB2000 spectrophotometer for spectral resolution. To minimize stray light and suppress the Rayleigh-scattered excitation component, a long-pass optical filter with a cut-off wavelength of 500 nm was installed in the detection path. This ensured that only the relevant PL signal was acquired, enhancing the signal-to-noise ratio. Environmental conditions were rigorously controlled throughout the *in-situ* PL measurements. The temperature was maintained at 25 °C, and the relative humidity was stabilized between 30–40%. All experiments were conducted in a dark environment to eliminate ambient light interference. These measurements significantly reduced external perturbations and ensured the reproducibility and accuracy of the photoluminescence data.

- 1. **Stability Testing Details**

The unencapsulated PSCs were illuminated under a 1-sun white light emitting diode (LED) lamp (SLS-LED-80A) in N_2_ atmosphere at room temperature to track the performance of PSCs at MPP. During the thermal stability test, given the poor thermal stability of Spiro-OMeTAD, PTAA is used as a substitute for Spiro-OMeTAD. Specifically, 15 mg PTAA with the addition of 5 μL Li-TFSI ((from 260 mg mL^-1^ stock acetonitrile solution) and 10 μL tBP was deposited by spin coating at 2000 rpm for 30 s.

# **Calculations**

All calculations were carried out using the Gaussian 09 software package ^[3]^. For the structural optimization and calculation of electrostatic potential surfaces (ESP), the B3LYP functional was employed in combination with the def 2-TZVP basis set, incorporating empirical dispersion correction GD3 (BJ). With the assistance of the Multiwfn code ^[4]^, the maximum value of φ (φ_max_) and the minimum value of φ (φ_min_) of the structure were determined. The first-principles calculations were conducted within the framework of the density functional theory, adopting the projector augmented plane - wave method as implemented in the Vienna ab initio simulation package ^[5]^. The generalized gradient approximation of the Perdew, Burke, and Ernzerhof (PBE) functional was utilized to describe the exchange - correlation potential ^[6]^. The long-range van der Waals interaction was accounted for by the DFT-D3 approach ^[7]^. The FAPbI_3_ (001) surface was constructed using a p (3×3) unit cell with a Pb-I termination. This unit cell consisted of 18 carbon (C) atoms, 90 hydrogen (H) atoms, 36 nitrogen (N) atoms, 27 lead (Pb) atoms, and 72 iodine (I) atoms. To prevent interactions between neighboring images, a vacuum spacing of over 16 Å was introduced in the z - direction. A plane-wave basis with a kinetic energy cutoff of 500 eV and the Monkhorst-Pack scheme ^[8]^ with a k - point grid spacing of 2π×0.04 Å⁻¹ were employed to ensure the convergence of the total energy. The convergence criteria for ionic and electronic optimizations were set as 0.02 eV/Å and 1×10^-5^ eV, respectively. The adsorption energy *E* was defined as: *E* = *E*_(A+B_*_)_* -(*E*_A_+*E*_B_), where *E*_(A+B)_ represents the energy of the molecule adsorbed on the FAPbI_3_ (001) surface; *E_B_* represents the energy of the molecule; and *E_A_* represents the energy of the FAPbI_3_ (001) surface.

1. Figures


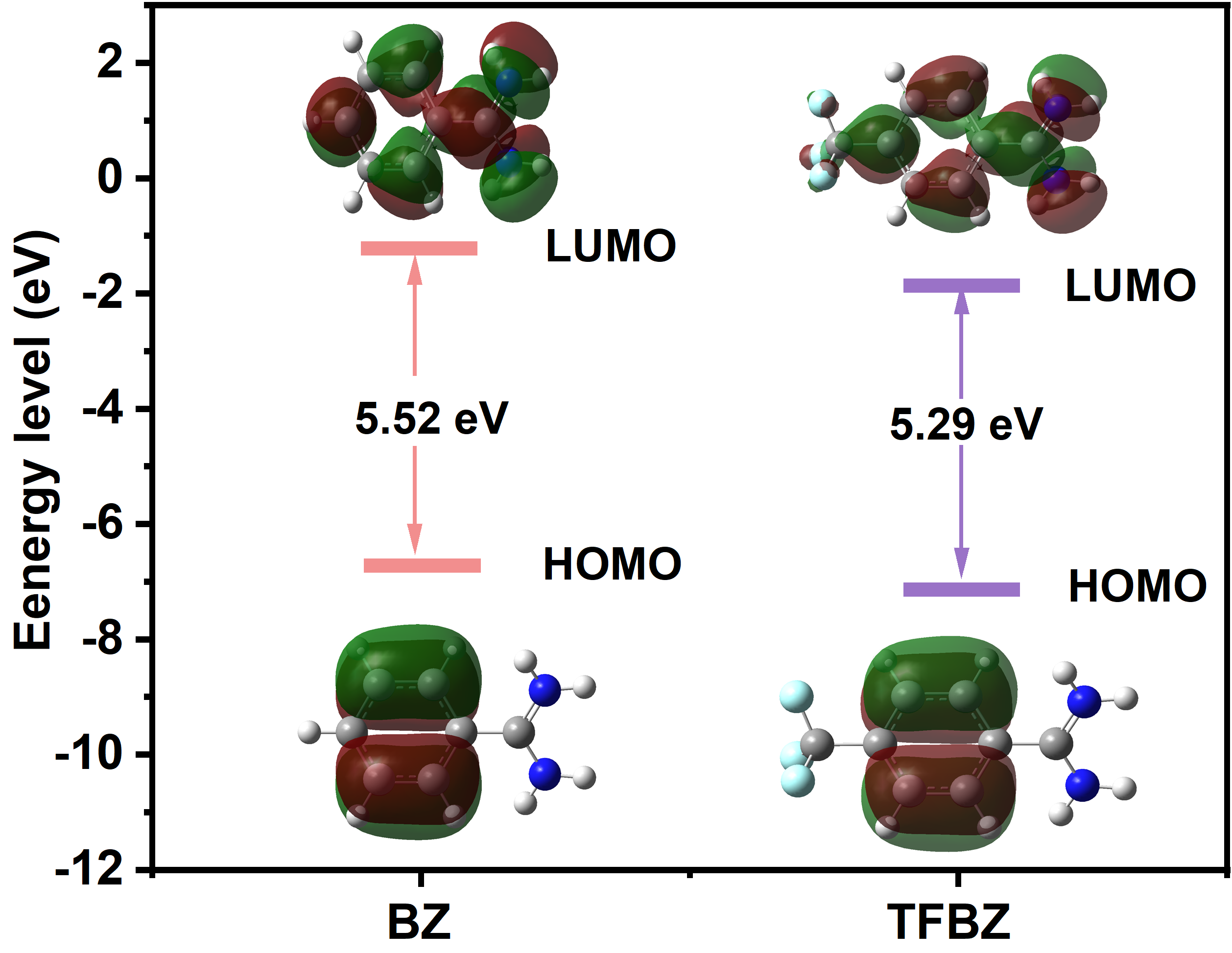


**Figure S1.** Frontier molecular orbital energy level diagram of BZ and TFBZ spacer cations by DFT calculations.


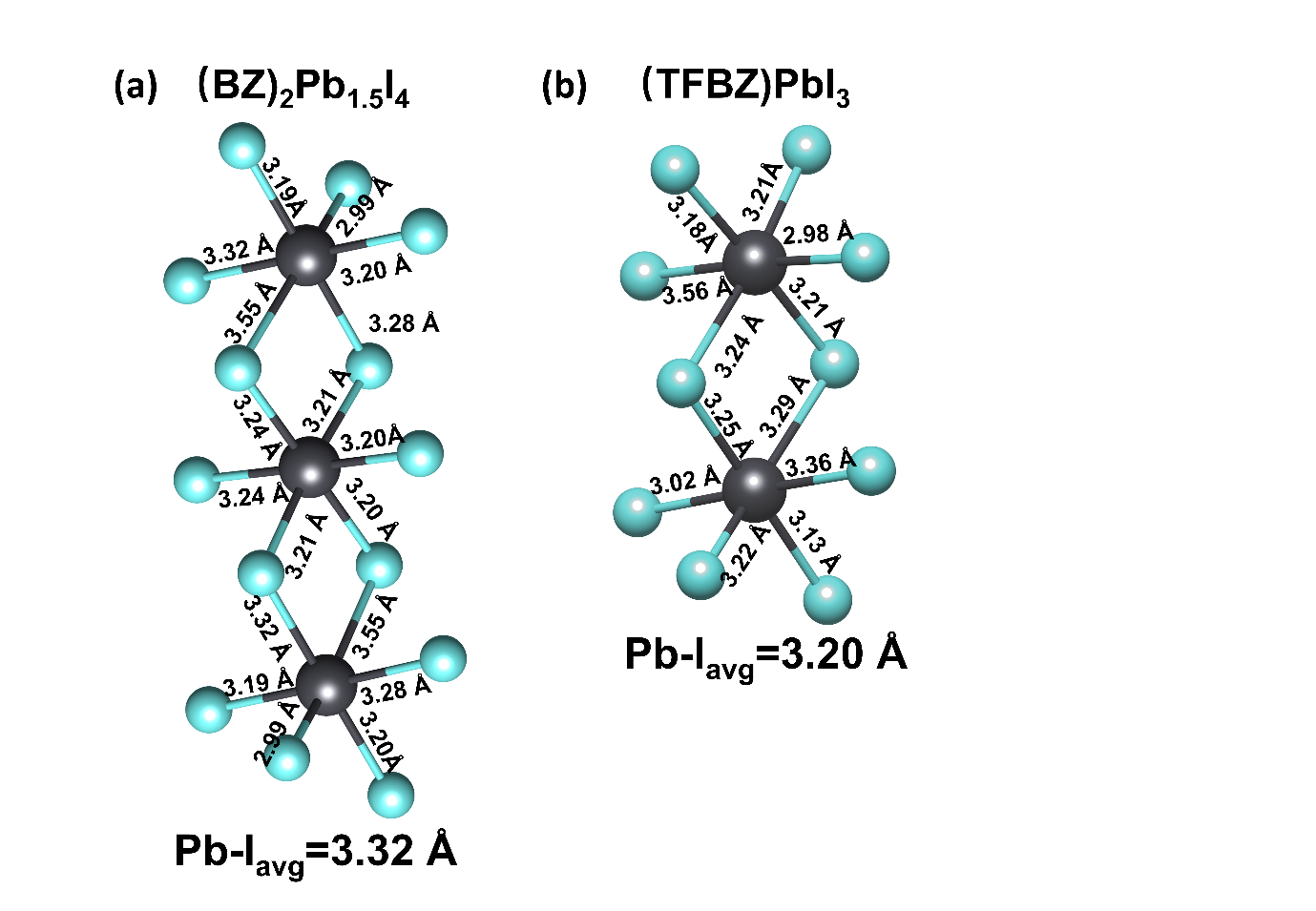


**Figure S2.** Average Pb-I bond lengths (Pb-I_avg_) in 1D (a) (BZ)_2_Pb_1.5_I_4_ and (b) (TFBZ)PbI_3_ single crystals.


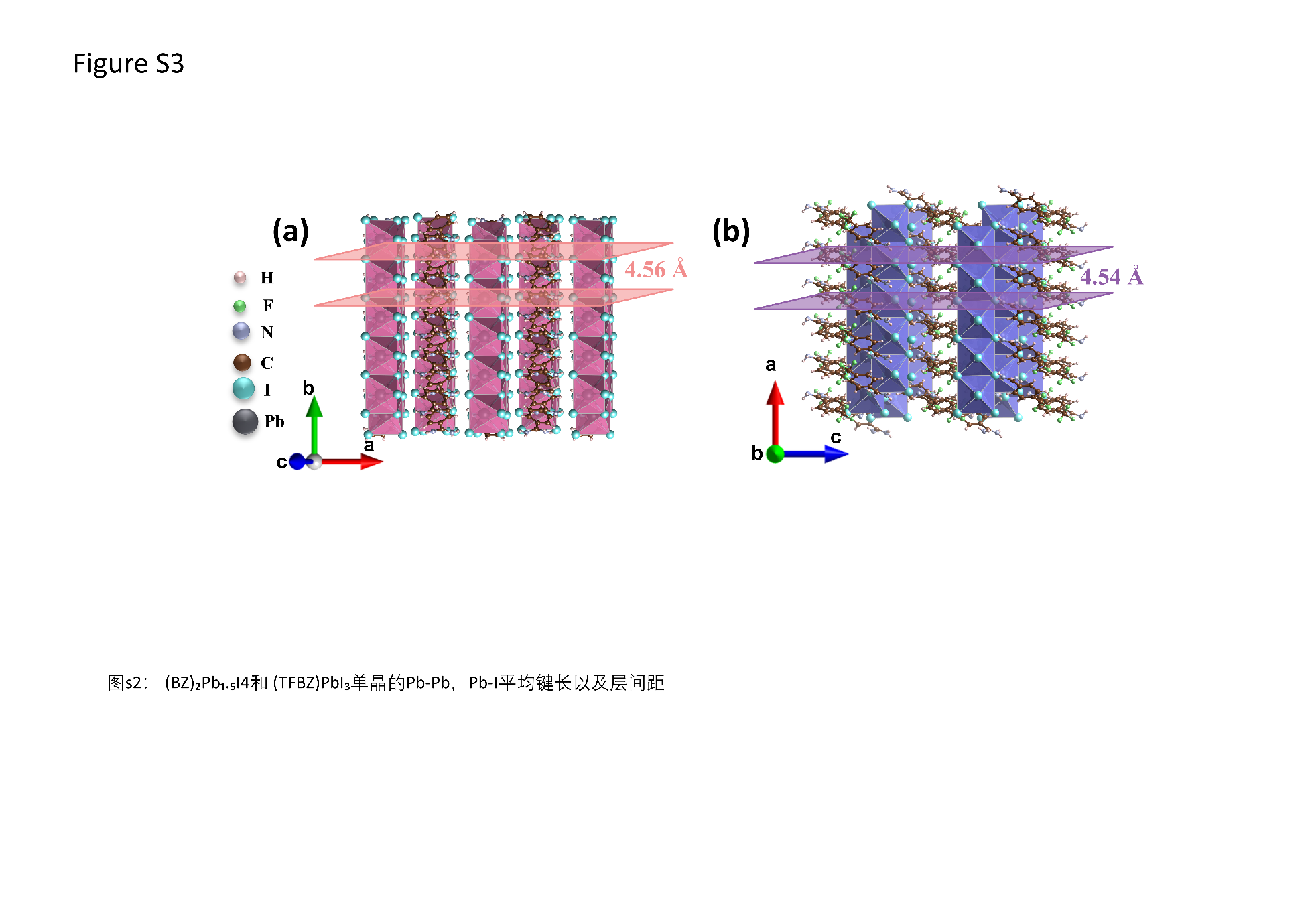


**Figure S3.** Interlayer spacing of 1D (a) (BZ)_2_Pb_1.5_I_4_ and (b) (TFBZ)PbI_3_ single crystals.


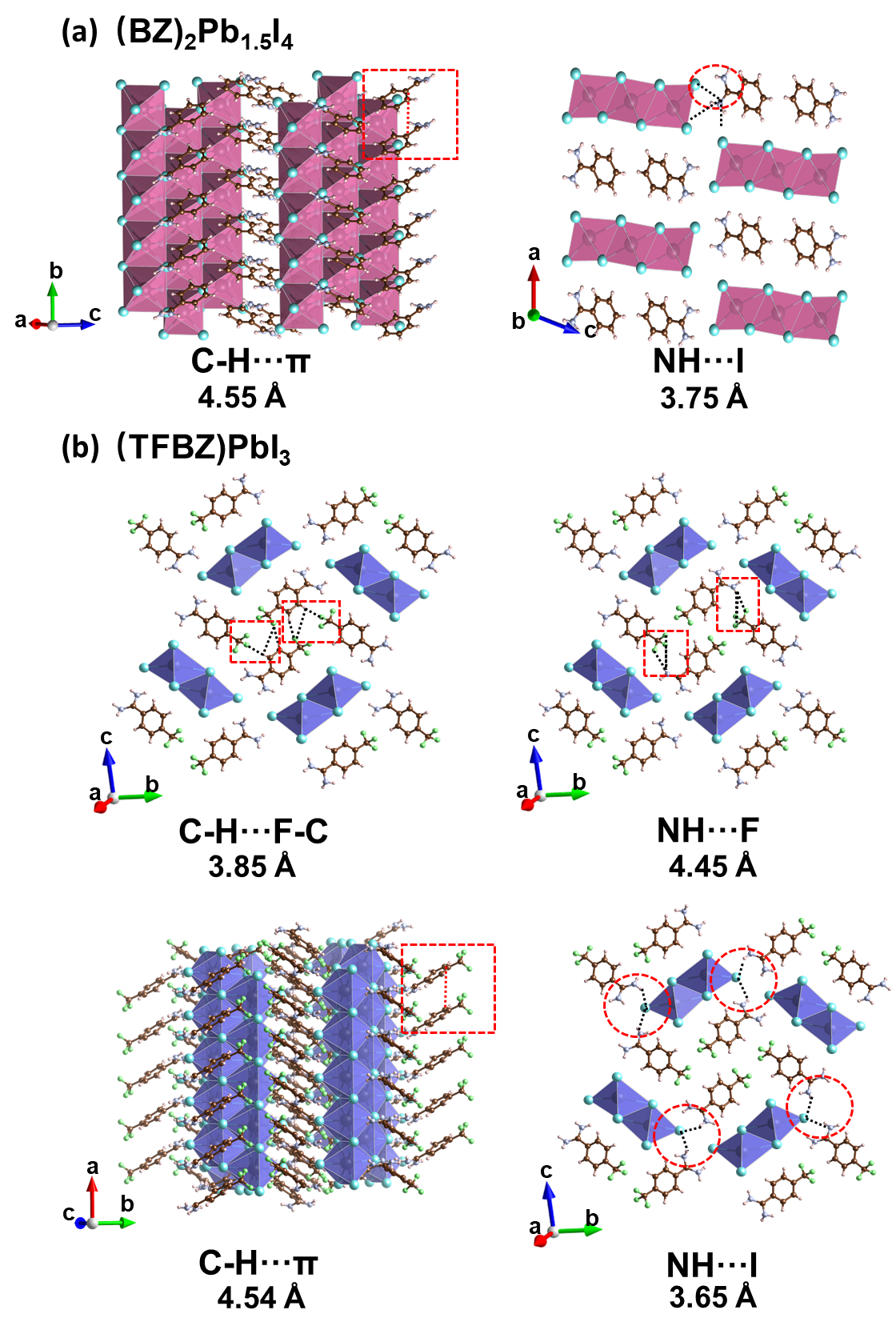


**Figure S4.** Schematic illustrations of the molecular structures and bonding modes in 1D (a) (BZ)_2_Pb_1.5_I_4_ and (b) (TFBZ)PbI_3_ single crystals.​


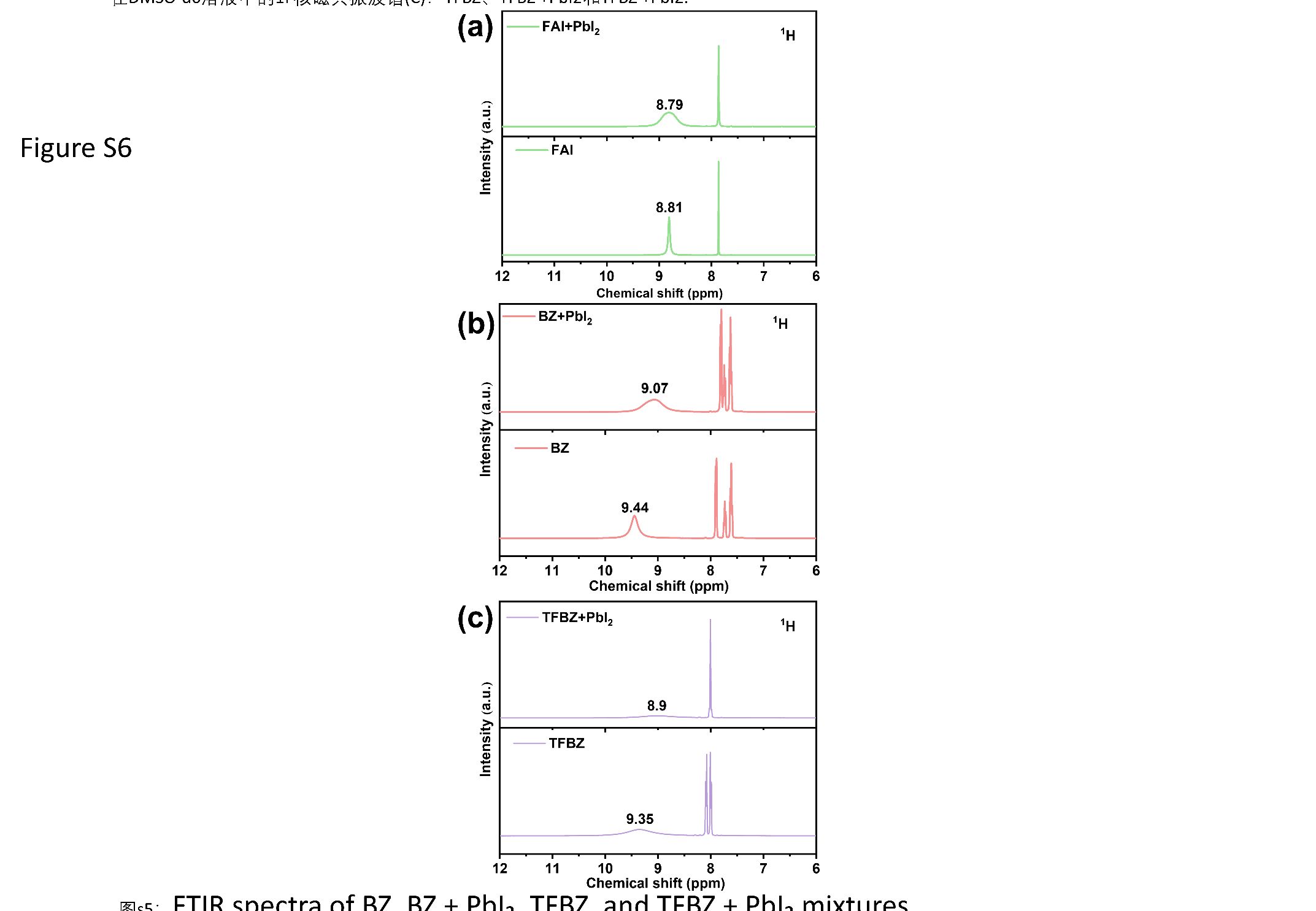


**Figure S5.** ¹H NMR spectra of (a) FAI and FAI+PbI_2_, (b) BZ and BZ+PbI₂, (c) TFBZ and TFBZ+PbI₂ ​(solvent: DMSO-d₆).


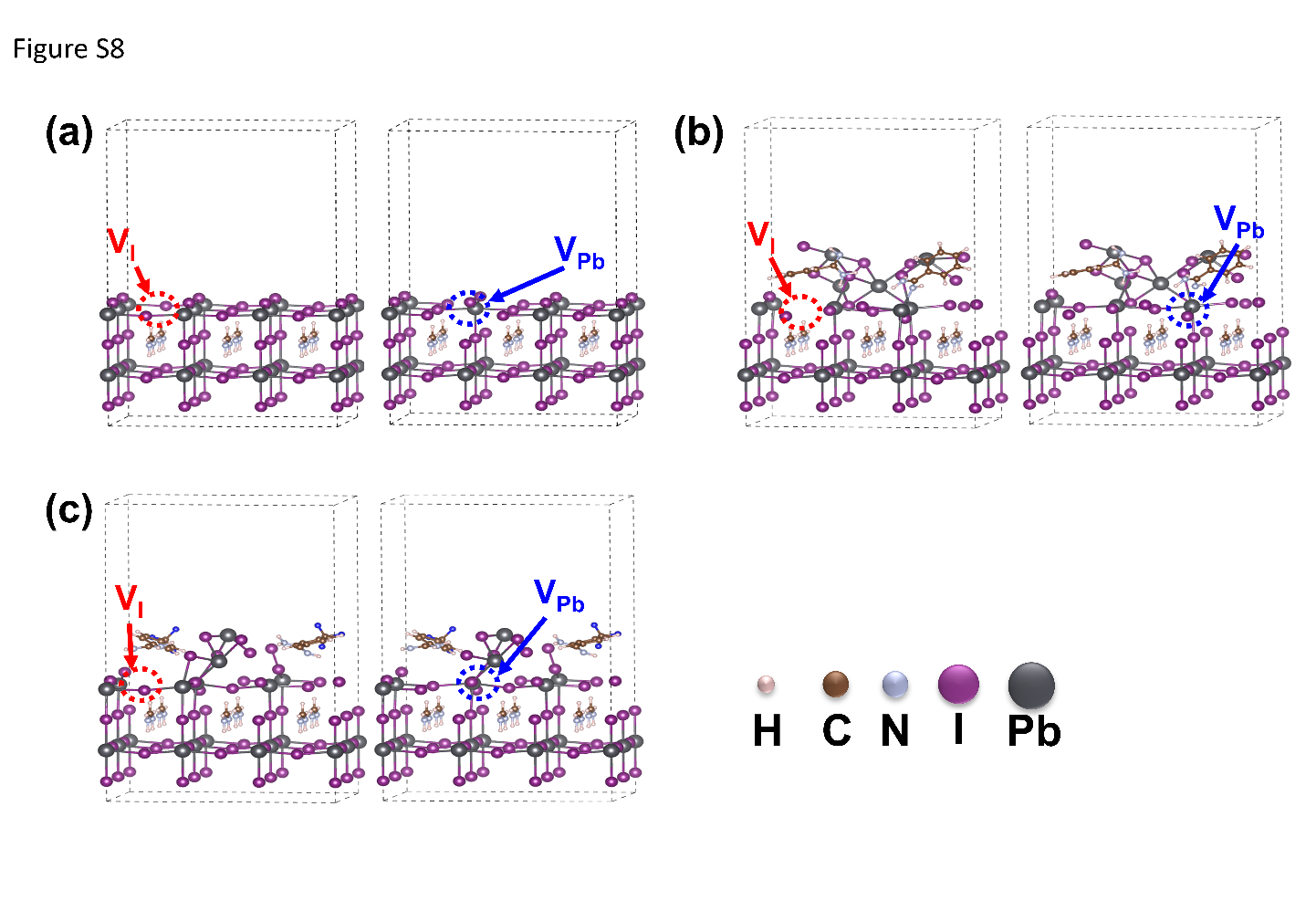


**Figure S6.** The schematic diagram of the defects of iodine vacancy (V_I_) and lead vacancy (V_Pb_) (a) at the Pb-I terminated surfaces for the control film as well as at the (b) 1D (BZ)_2_Pb_1.5_I_4_/3D and (c) 1D (TFBZ)PbI_3_/3D perovskite heterostructure interfaces.


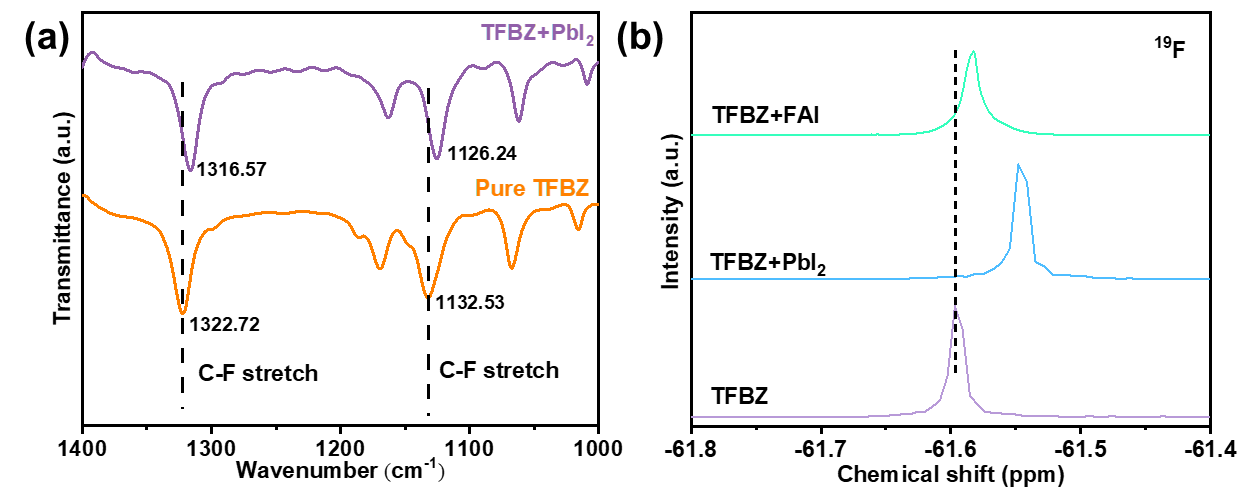


**Figure S7**. (a) FTIR spectra of the TFBZ and TFBZ+PbI_2_. (b) ^19^F NMR spectra of TFBZ, TFBZ+PbI₂ and TFBZ+FAI. (solvent: deuterated chloroform).


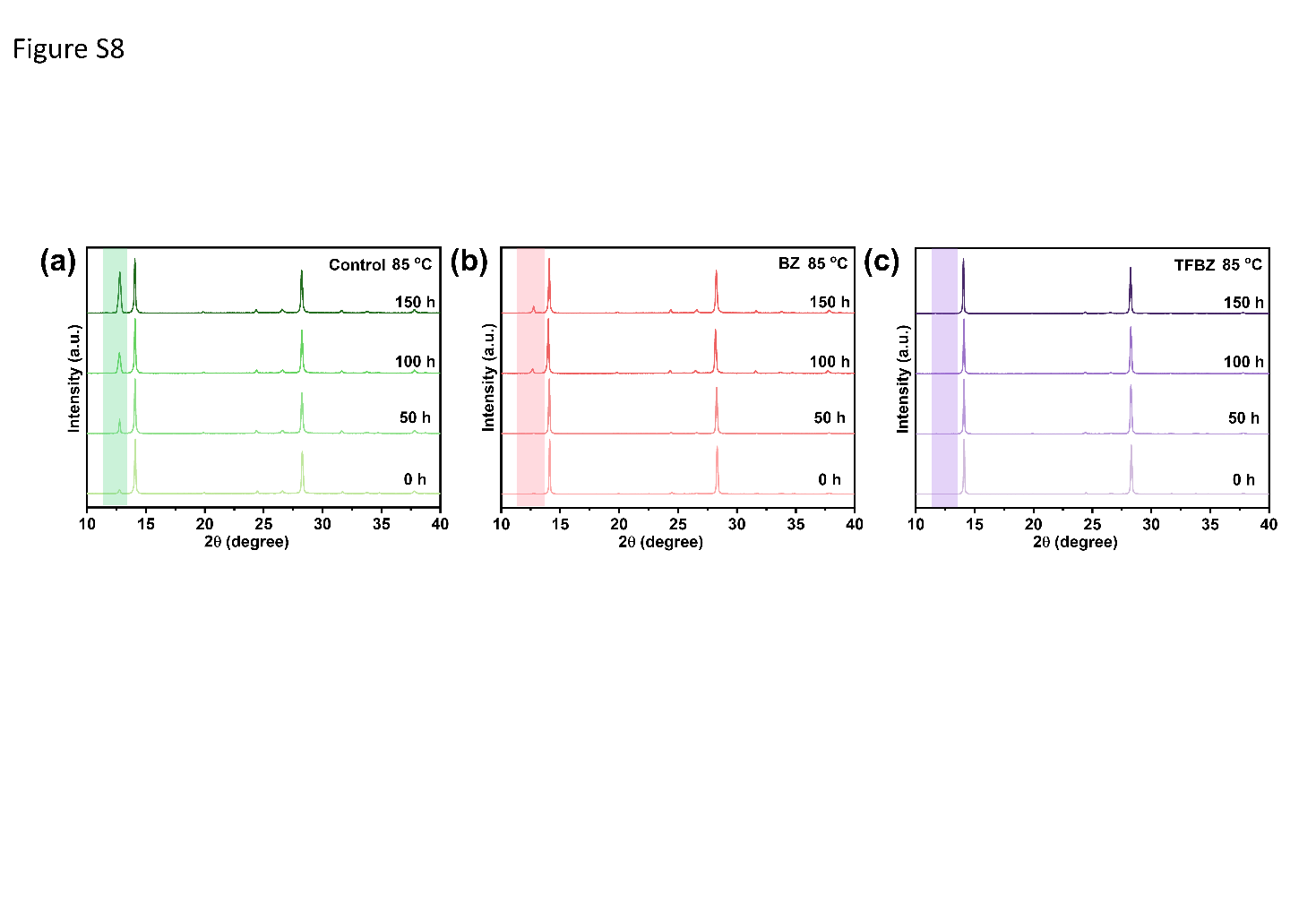


Figure S8. XRD patterns of a) Control, b) BZ-based and c) TFBZ-based perovskite films after being heated at 85 °C in N_2_ for 150 h.


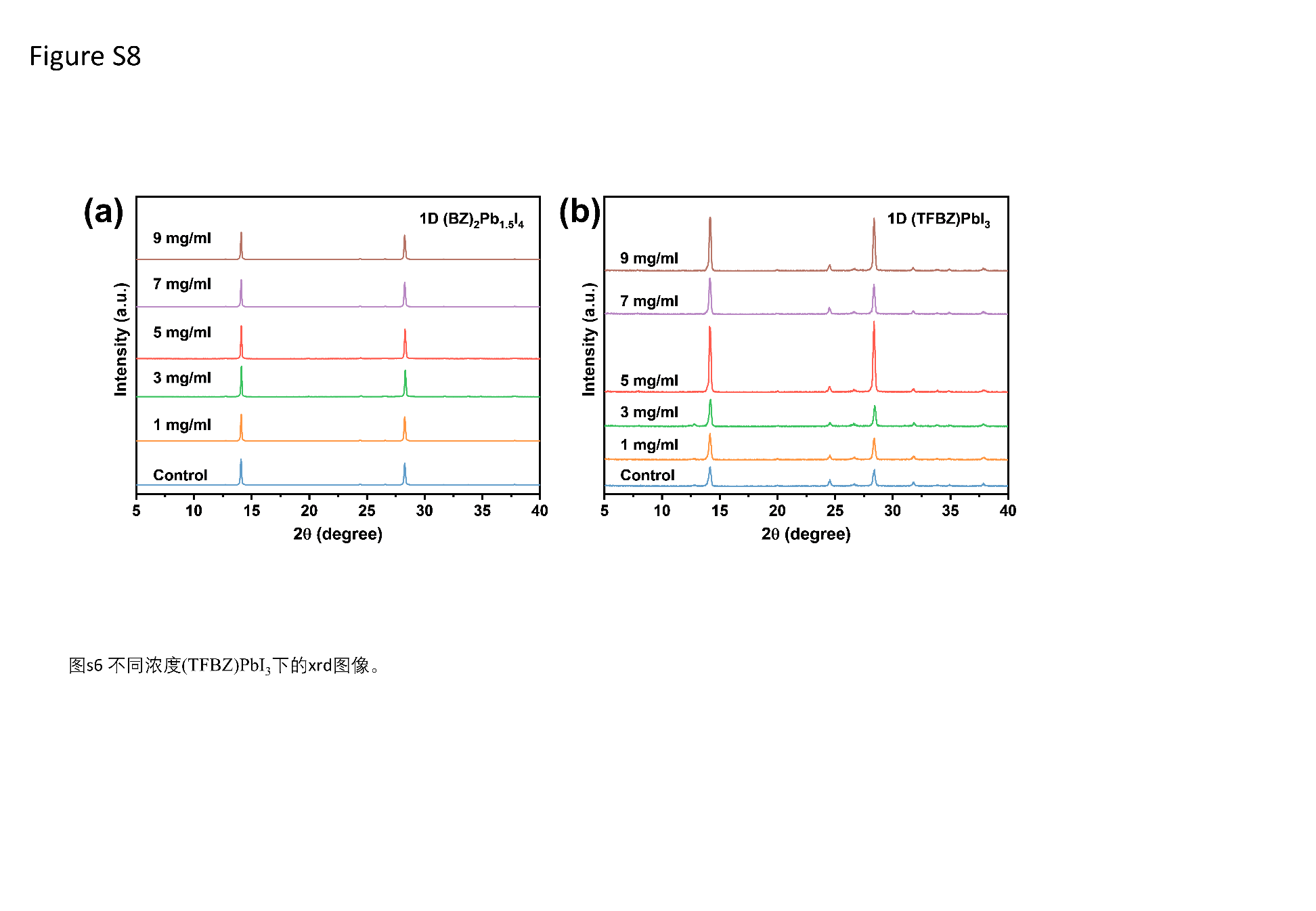


**Figure S9.** XRD patterns of the perovskite films with different concentration of 1D (a) (BZ)_2_Pb_1.5_I_4_ and (b) (TFBZ)PbI_3_ single crystals.


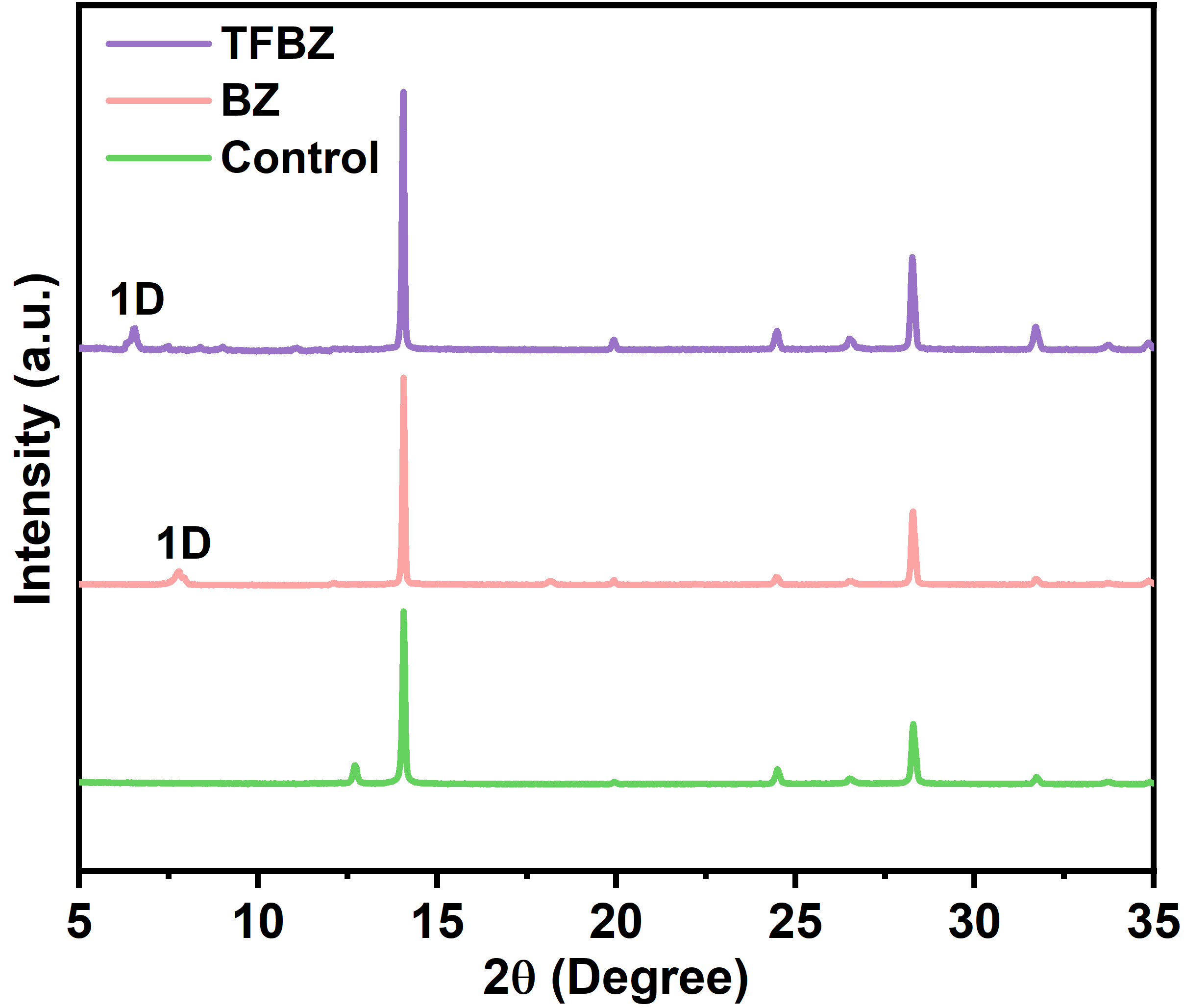


**Figure S10.** XRD patterns of the control, BZ-based and TFBZ-based 1D/3D perovskite films. The concentration of the added 1D perovskite in both 1D/3D perovskite film was 20 mg%.


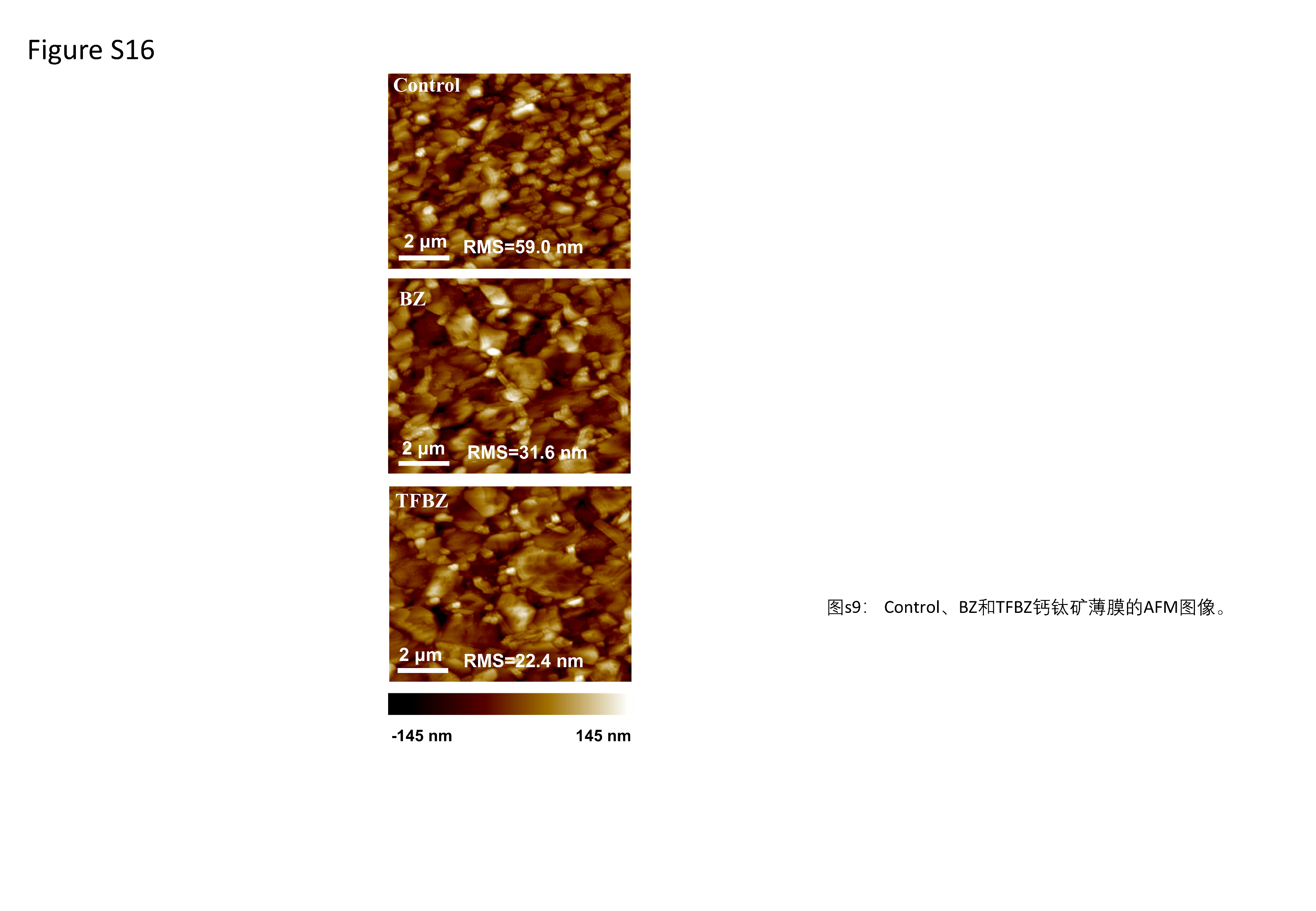


**Figure S11.** AFM images of control, BZ-treated and TFBZ-treated 1D/3D perovskite films.


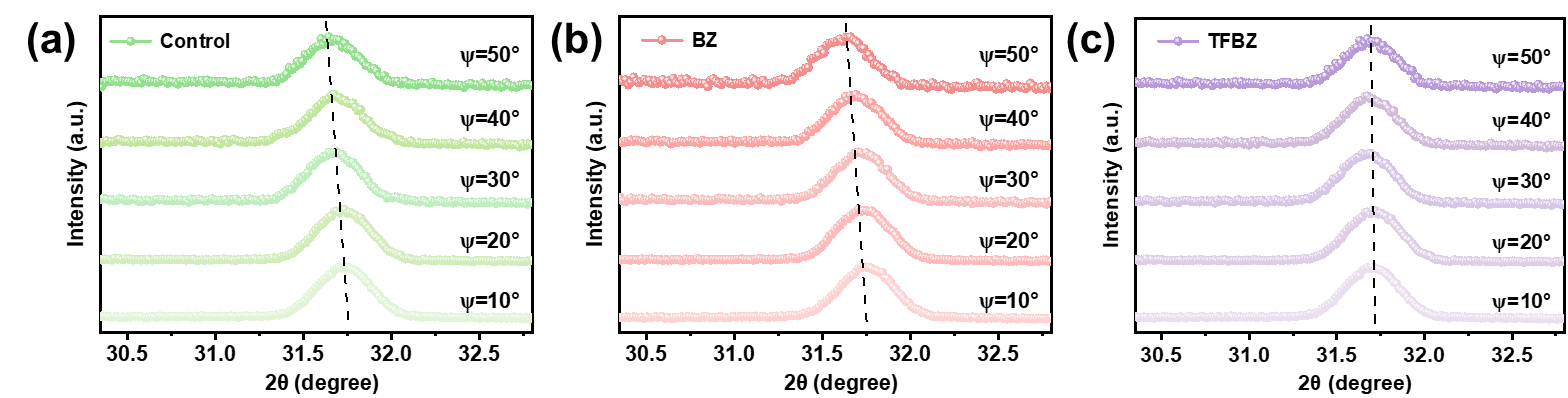


**Figure S12.** The GIXRD patterns measured in the condition of φ=1^o^ as a function of instrumental ψ values from 0^o^ to 50^o^.


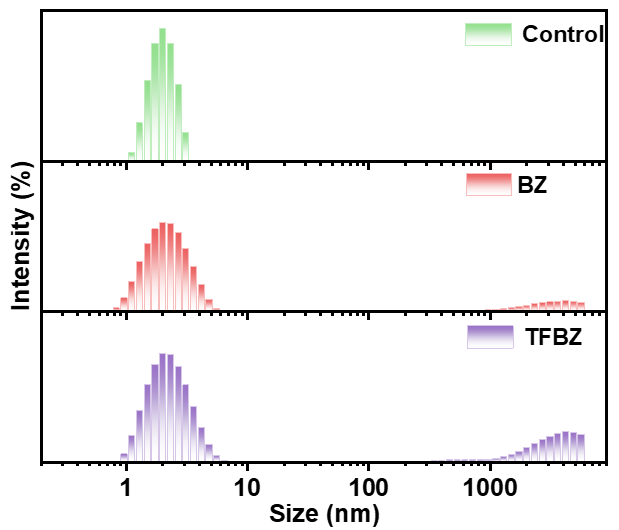


**Figure S13.** DLS profiles of the control, BZ-treated and TFBZ-treated perovskite precursor solutions.

**
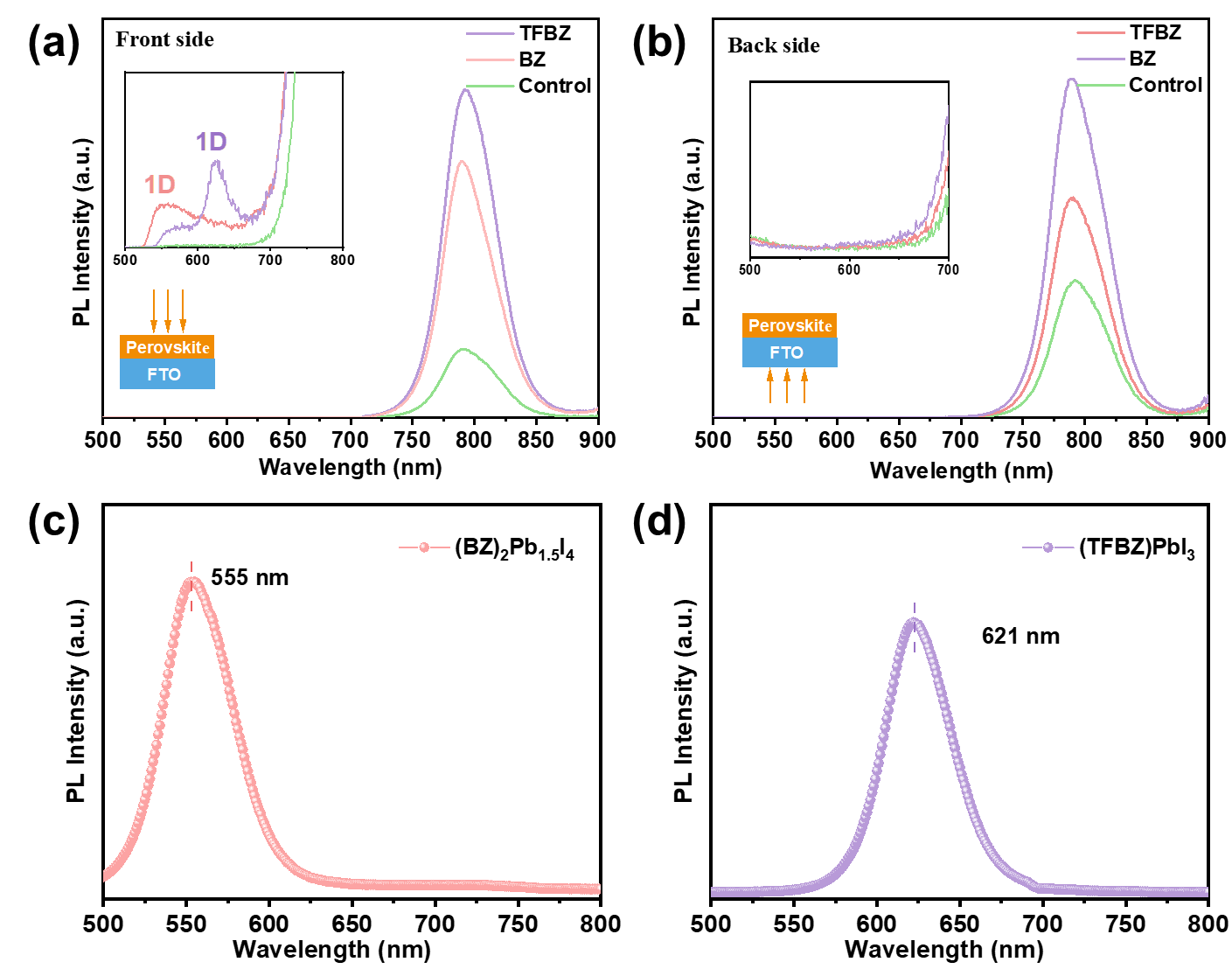
**

**Figure S14.** Steady-state PL spectra of the perovskite films under (a) front and (b) back incident directions. Steady-state PL spectra of the 1D (c) (BZ)_2_Pb_1.5_I_4_ and (d) (TFBZ)PbI_3_ single crystals.


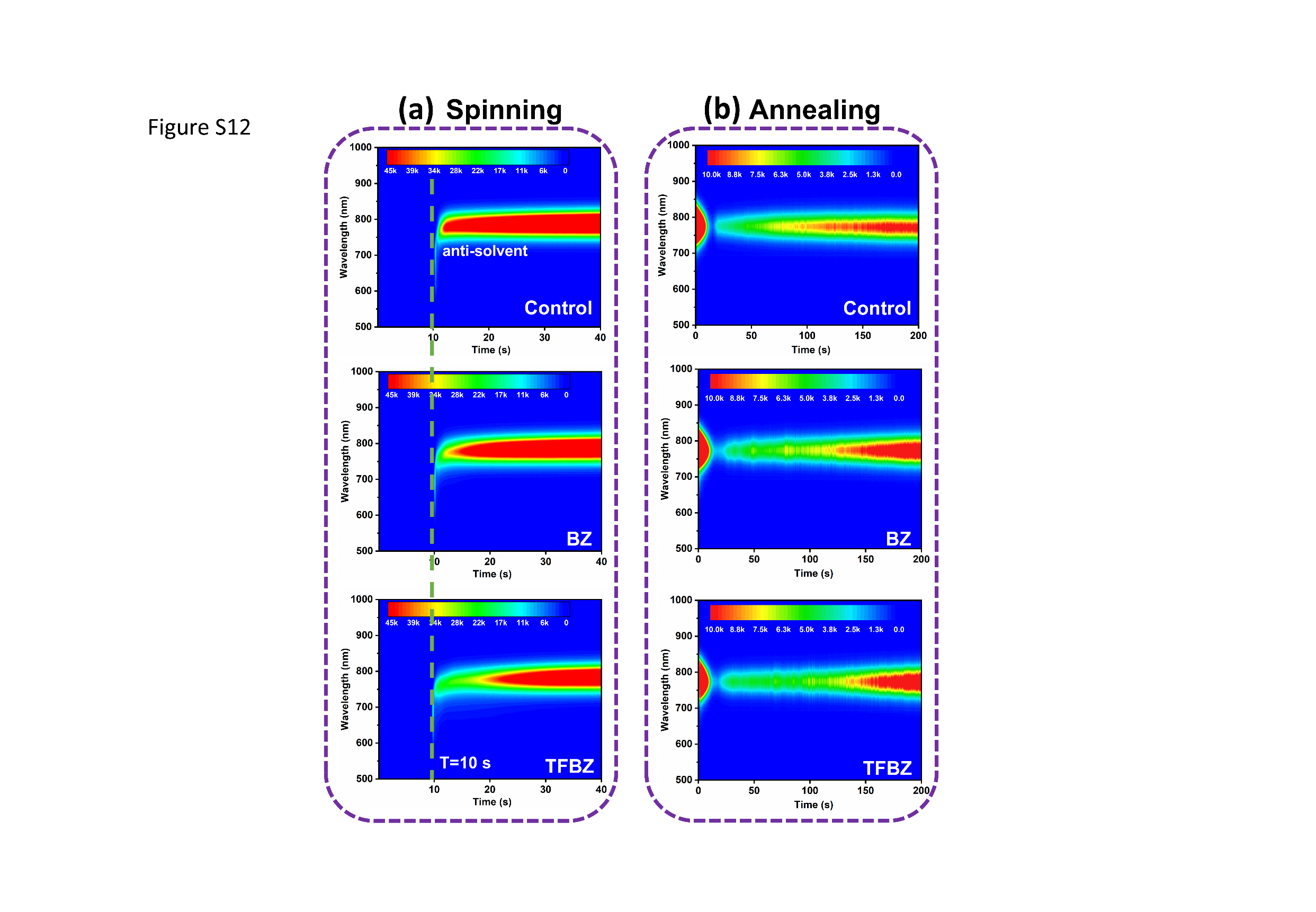


**Figure S15.** In situ PL spectra of the control, BZ-treated and TFBZ-treated 1D/3D perovskite films during (a) spin coating and (b) annealing.


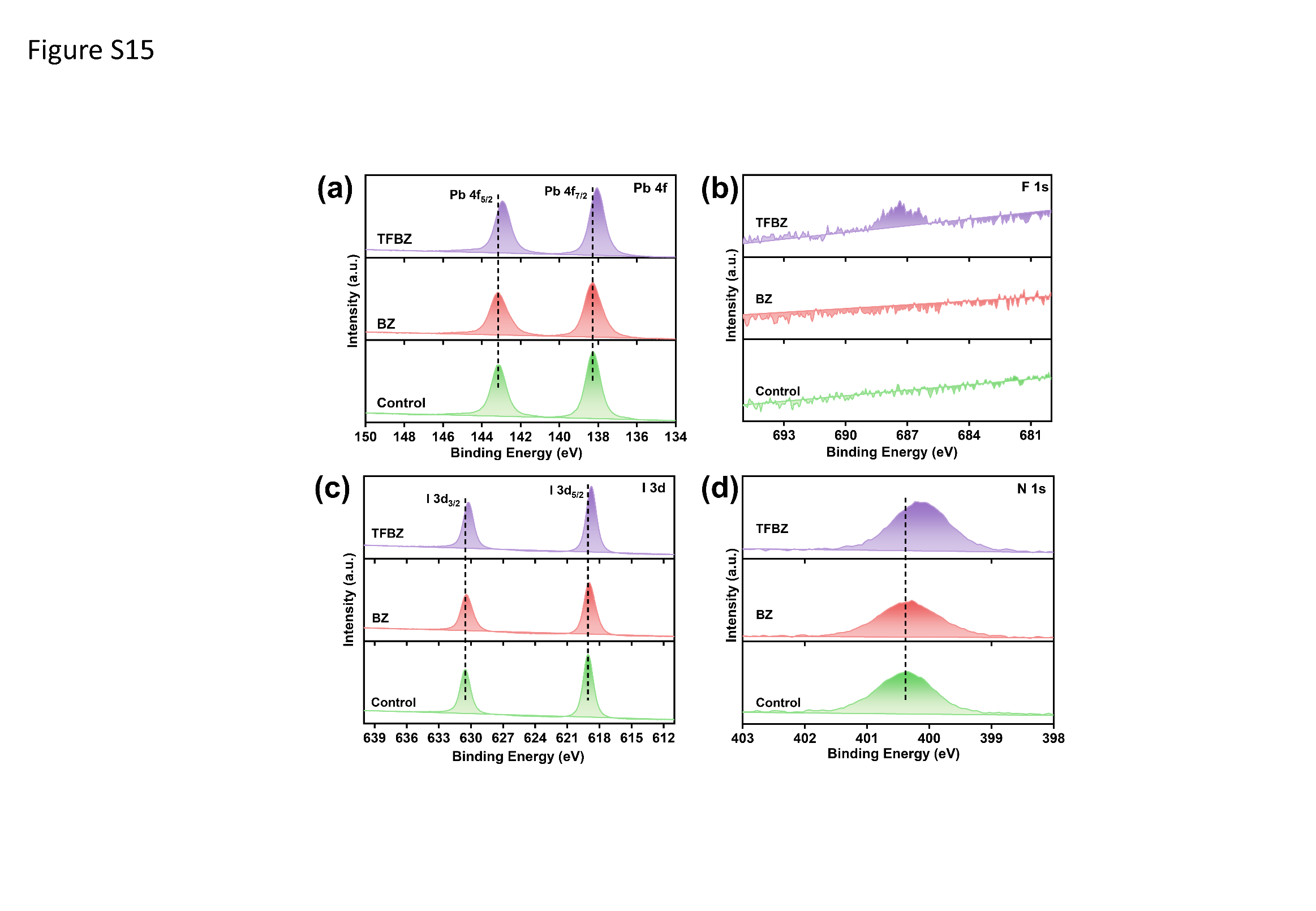


**Figure S16**. High-resolution (a) Pb 4f, (b) F1s, (c) I 3d and (d) N 1s XPS spectra of control, BZ-treated, and TFBZ-treated 1D/3D perovskite films.


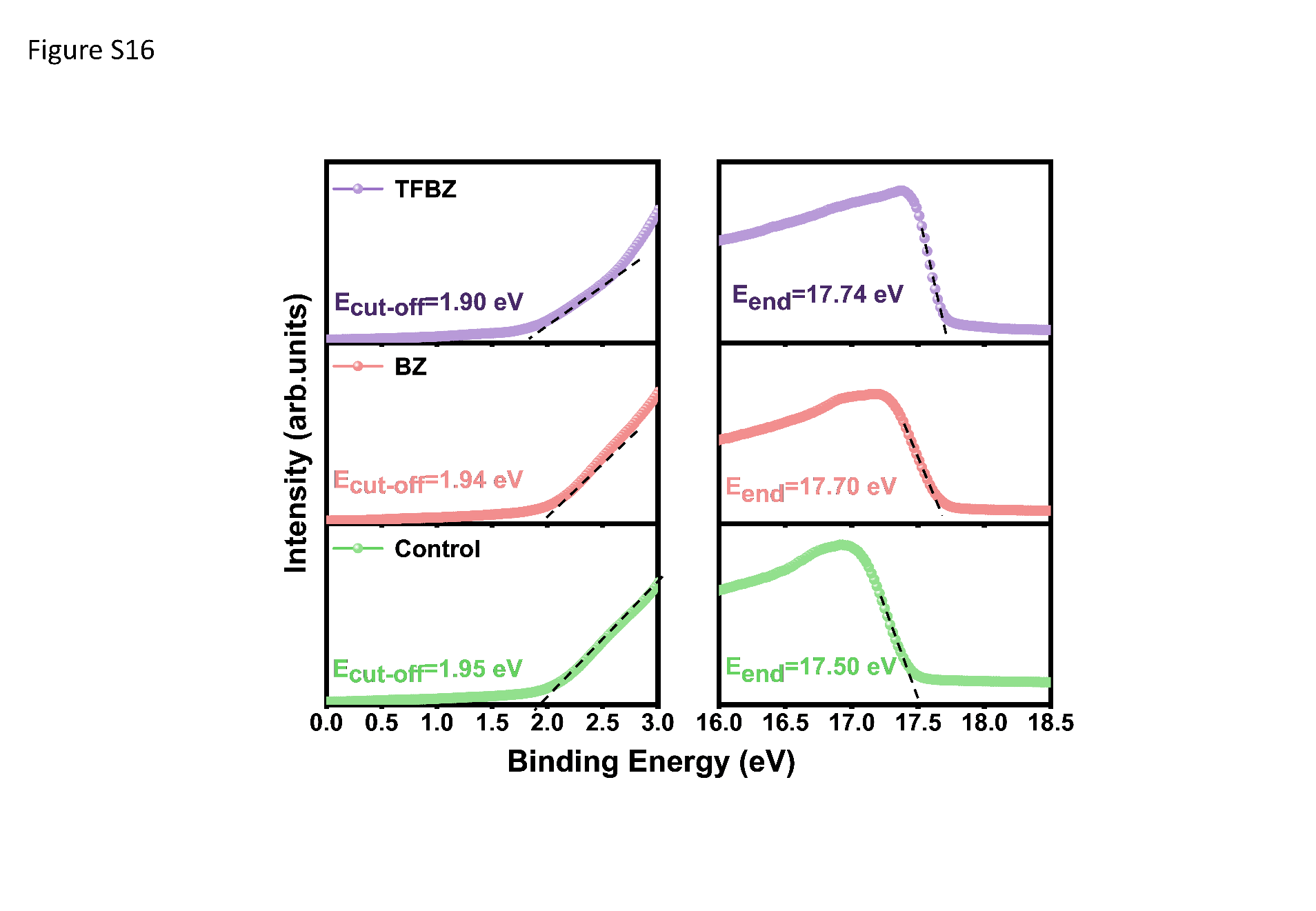


**Figure S17.** UPS spectra of the control, BZ-treated and TFBZ-treated 1D/3D perovskite films.


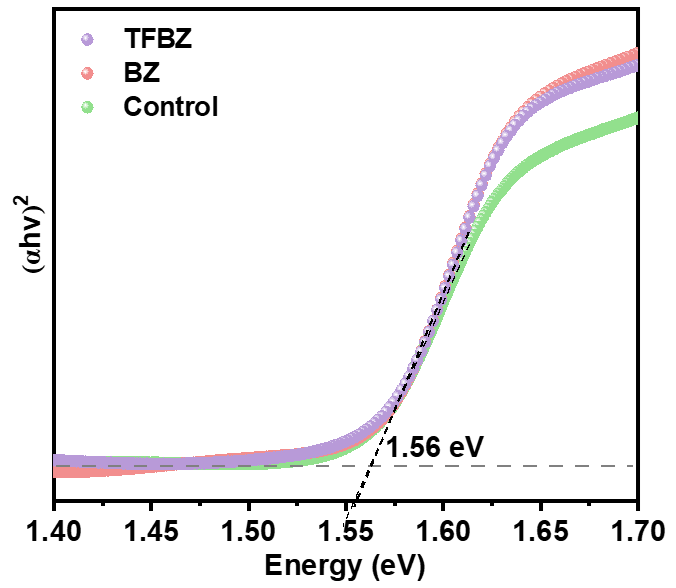


**Figure S18.** UV-vis absorption spectra of the control, BZ-treated and TFBZ-treated 1D/3D perovskite films.


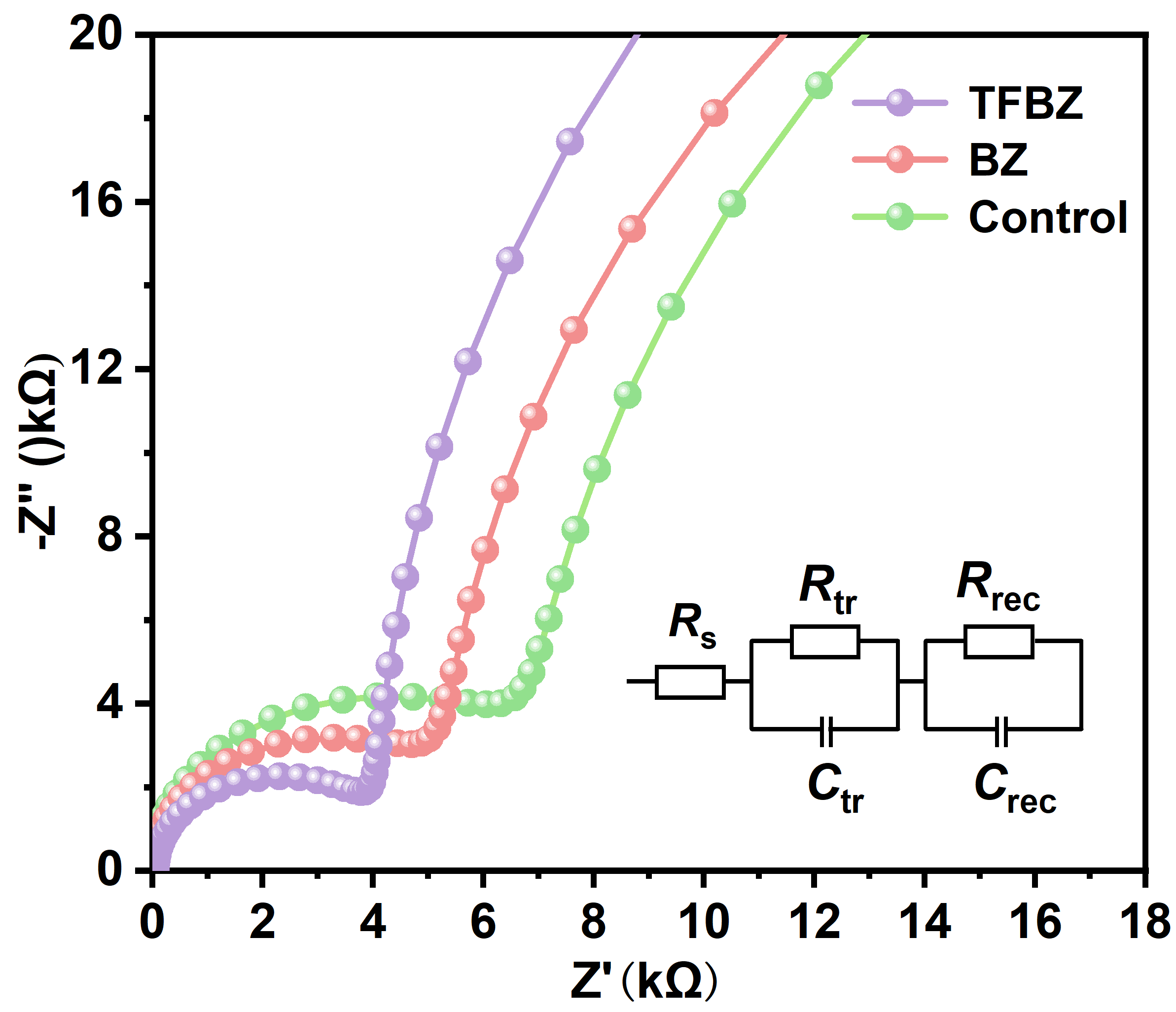


**Figure S19.** Nyquist plots of the control, BZ-treated, and TFBZ-treated PSCs. The inset shows the equivalent circuit model.


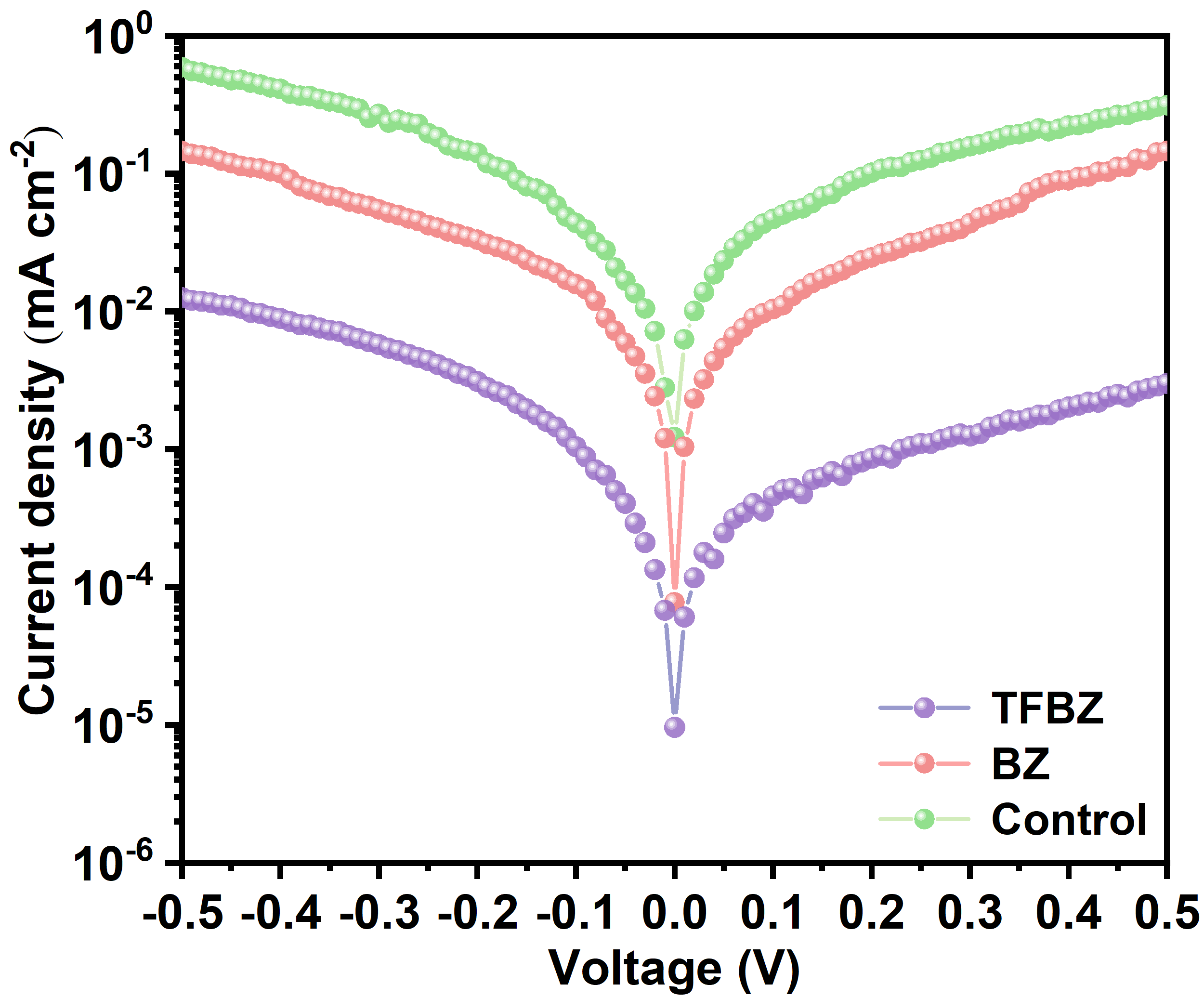


**Figure S20.** Dark *J*–*V* curves of the control, BZ-treated, and TFBZ-treated PSCs.

**
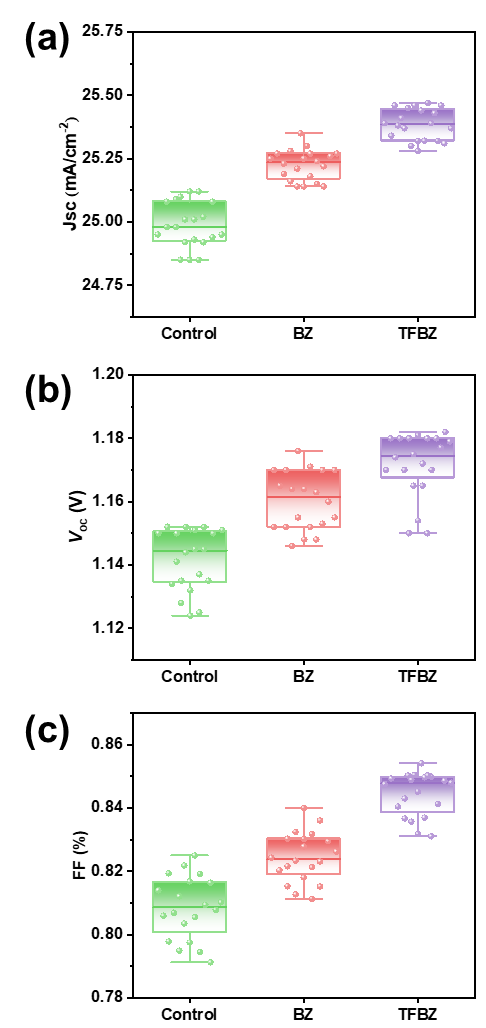
**

**Figure S21.** Statistical distribution of (a) FF, (b) *J*_sc_, and (c) *V*_oc_ from 20 independent cells.

**Figure S22.** PCE evolution of the unencapsulated 1D/3D and control devices under ambient condition (40% RH).

# **Tables**

**Table S1.** Crystal data and structure refinement for 1D (TFBZ)PbI_3_ single crystals.

| Empirical formula | C_16_H_14_F_6_I_6_N_4_Pb_2_ |
| --- | --- |
| Formula weight | 1552.09 |
| Temperature [K] | 240.0 |
| Crystal system | monoclinic |
| Space group (number) | $P2_{1}/c$ (14) |
| *a* [Å] | 4.5435(6) |
| *b* [Å] | 25.194(4) |
| *c* [Å] | 26.838(4) |
| α [°] | 90 |
| β [°] | 90 |
| γ [°] | 90 |
| Volume [Å^3^] | 3072.2(7) |
| *Z* | 4 |
| *ρ*_calc_ [gcm^−3^] | 3.356 |
| *μ* [mm^−1^] | 17.025 |
| *F*(000) | 2696 |
| Crystal size [mm^3^] | 0.01×0.02×0.03 |
| Crystal colour | clear light yellow |
| Crystal shape | block |
| Radiation | Mo*K_α_* (λ=0.71073 Å) |
| 2θ range [°] | 3.44 to 50.87 (0.83 Å) |
| Index ranges | −5 ≤ h ≤ 5 −30 ≤ k ≤ 30 −32 ≤ l ≤ 32 |
| Reflections collected | 19864 |
| Independent reflections | 5642  *R*_int_ = 0.0630 *R*_sigma_ = 0.0656 |
| Completeness to θ = 25.242° | 99.5 % |
| Data / Restraints / Parameters | 5642 / 1 / 313 |
| Absorption correction T_min_/T_max_ (method) | 0.5348 / 0.7452  (none) |
| Goodness-of-fit on *F*^2^ | 1.100 |
| Final *R* indexes  [*I*≥2σ(*I*)] | *R*_1_ = 0.0559 w*R*_2_ = 0.1178 |
| Final *R* indexes  [all data] | *R*_1_ = 0.0885 w*R*_2_ = 0.1315 |
| Largest peak/hole [eÅ^−3^] | 3.46/−1.57 |

**Table S1.** Atomic coordinates and *U_eq_* [Å^2^] for 1D (BZ)_2_Pb_1.5_I_4_ single crystals.

| **Atom** | ***x*** | ***y*** | ***z*** | ***U*_eq_** |
| --- | --- | --- | --- | --- |
| Pb1 | 1.16192(16) | 0.20037(3) | 0.24567(3) | 0.0327(2) |
| Pb2 | 0.72505(17) | 0.07213(3) | 0.35965(3) | 0.0335(2) |
| I1 | 1.2118(3) | 0.07076(5) | 0.27319(4) | 0.0322(3) |
| I2 | 0.7432(3) | −0.04559(5) | 0.34932(5) | 0.0415(3) |
| I3 | 1.1912(3) | 0.31810(5) | 0.22494(5) | 0.0405(3) |
| I4 | 1.6606(3) | 0.20994(5) | 0.33165(5) | 0.0373(3) |
| I5 | 0.2283(3) | 0.06804(7) | 0.44328(5) | 0.0541(4) |
| I6 | 1.6480(3) | 0.18636(5) | 0.16434(5) | 0.0394(3) |
| F1 | −0.129(4) | 0.5903(7) | 0.4086(7) | 0.100(6) |
| F4 | 0.255(4) | 0.5985(7) | 0.4466(7) | 0.110(7) |
| F6 | −0.066(5) | 0.5414(7) | 0.4690(7) | 0.140(10) |
| N1 | 0.742(4) | 0.4266(7) | 0.2637(6) | 0.044(4) |
| H1A | 0.842622 | 0.405153 | 0.244860 | 0.052 |
| H1B | 0.702557 | 0.458585 | 0.253480 | 0.052 |
| N3 | 0.711(5) | 0.3634(6) | 0.3216(7) | 0.058(6) |
| C1 | 0.648(4) | 0.4105(7) | 0.3082(8) | 0.038(5) |
| C4 | 0.500(4) | 0.4503(7) | 0.3387(7) | 0.033(4) |
| C7 | 0.204(5) | 0.5278(9) | 0.3482(8) | 0.049(6) |
| H7 | 0.111506 | 0.557549 | 0.333986 | 0.059 |
| C8 | 0.526(5) | 0.4454(7) | 0.3899(7) | 0.045(5) |
| H8 | 0.644137 | 0.418889 | 0.404176 | 0.054 |
| C9 | 0.328(4) | 0.4905(8) | 0.3188(8) | 0.042(5) |
| H9 | 0.295940 | 0.491703 | 0.284232 | 0.050 |
| C10 | 0.215(4) | 0.5220(8) | 0.4002(7) | 0.035(5) |
| C13 | 0.084(6) | 0.5622(10) | 0.4331(10) | 0.062(7) |
| C16 | 0.364(5) | 0.4824(9) | 0.4208(8) | 0.048(6) |
| H16 | 0.365158 | 0.478314 | 0.455575 | 0.058 |
| F2 | 0.409(6) | 0.3544(8) | 0.4808(7) | 0.148(10) |
| F3 | 0.374(7) | 0.3095(6) | 0.4188(8) | 0.180(14) |
| F5 | 0.046(4) | 0.3534(9) | 0.4430(12) | 0.183(14) |
| N2 | −0.306(4) | 0.1624(8) | 0.6187(6) | 0.048(5) |
| N4 | −0.256(4) | 0.1053(7) | 0.5547(6) | 0.049(5) |
| H4A | −0.333953 | 0.080674 | 0.573191 | 0.059 |
| H4B | −0.201645 | 0.098042 | 0.524355 | 0.059 |
| C2 | 0.132(4) | 0.2780(8) | 0.4877(8) | 0.039(5) |
| C3 | 0.070(4) | 0.2359(8) | 0.5651(7) | 0.040(5) |
| H3A | 0.106020 | 0.234206 | 0.599599 | 0.048 |
| C5 | −0.030(5) | 0.2382(8) | 0.4652(7) | 0.042(5) |
| H5 | −0.065900 | 0.240080 | 0.430768 | 0.050 |
| C6 | −0.220(5) | 0.1537(8) | 0.5727(7) | 0.039(5) |
| C11 | 0.234(5) | 0.3221(9) | 0.4588(9) | 0.049(6) |
| C12 | −0.140(5) | 0.1965(7) | 0.4915(7) | 0.038(5) |
| H12 | −0.245777 | 0.169195 | 0.475650 | 0.046 |
| C14 | −0.092(5) | 0.1958(8) | 0.5413(7) | 0.043(5) |
| C15 | 0.176(4) | 0.2778(9) | 0.5374(7) | 0.043(5) |
| H15 | 0.278263 | 0.305813 | 0.552834 | 0.052 |
| H3 | 0.62(5) | 0.351(9) | 0.344(8) | 0.052 |
| H2 | −0.34(5) | 0.203(8) | 0.618(8) | 0.052 |

**Table S1.** Crystal data and structure refinement for 1D (BZ)_2_Pb_1.5_I_4_ single crystals.

| **Empirical formula** | **C_7_H_8_I_4_N_2_Pb_1.50_** |
| --- | --- |
| Formula weight | 938.54 |
| Temperature [K] | 240.0 |
| Crystal system | monoclinic |
| Space group (number) | $C2/c$ (15) |
| *a* [Å] | 24.4645(16) |
| *b* [Å] | 4.5540(3) |
| *c* [Å] | 32.047(2) |
| α [°] | 90 |
| β [°] | 111.514(2) |
| γ [°] | 90 |
| Volume [Å^3^] | 3321.6(4) |
| *Z* | 8 |
| *ρ*_calc_ [gcm^−3^] | 3.754 |
| *μ* [mm^−1^] | 22.614 |
| *F*(000) | 3192 |
| Crystal size [mm^3^] | 0.01×0.02×0.03 |
| Crystal colour | clear light colourless |
| Crystal shape | block |
| Radiation | Mo*K_α_* (λ=0.71073 Å) |
| 2θ range [°] | 5.24 to 50.77 (0.83 Å) |
| Index ranges | −29 ≤ h ≤ 28 −5 ≤ k ≤ 5 −38 ≤ l ≤ 38 |
| Reflections collected | 11871 |
| Independent reflections | 3044  *R*_int_ = 0.0744 *R*_sigma_ = 0.0721 |
| Completeness to  θ = 25.242° | 99.6 % |
| Data / Restraints / Parameters | 3044 / 0 / 133 |
| Absorption correction T_min_/T_max_ (method) | 0.2475 / 0.7452  (Not applied) |
| Goodness-of-fit on *F*^2^ | 1.048 |
| Final *R* indexes  [*I*≥2σ(*I*)] | *R*_1_ = 0.0498 w*R*_2_ = 0.1245 |
| Final *R* indexes  [all data] | *R*_1_ = 0.0746 w*R*_2_ = 0.1404 |
| Largest peak/hole [eÅ^−3^] | 3.21/−3.29 |

**Table S1.** Atomic coordinates and *U_eq_* [Å^2^] for 1D (BZ)_2_Pb_1.5_I_4_ single crystals.

| **Atom** | ***x*** | ***y*** | ***z*** | ***U*_eq_** |
| --- | --- | --- | --- | --- |
| Pb1 | 0.500000 | 1.19429(16) | 0.250000 | 0.0326(2) |
| Pb2 | 0.54505(2) | 0.73060(11) | 0.39289(2) | 0.0335(2) |
| I1 | 0.60295(4) | 1.22383(19) | 0.34702(3) | 0.0332(3) |
| I2 | 0.66277(4) | 0.7373(2) | 0.46722(3) | 0.0404(3) |
| I3 | 0.49477(5) | 0.23235(19) | 0.43873(4) | 0.0425(3) |
| I4 | 0.56631(4) | 1.6880(2) | 0.21543(4) | 0.0368(3) |
| N1 | 0.8655(6) | 1.271(3) | 0.4487(5) | 0.062(4) |
| H1 | 0.885912 | 1.232373 | 0.432027 | 0.075 |
| N2 | 0.7671(5) | 1.244(2) | 0.4342(5) | 0.047(4) |
| H2A | 0.775039 | 1.151486 | 0.459412 | 0.057 |
| H2B | 0.730838 | 1.280230 | 0.417191 | 0.057 |
| C1 | 0.8241(7) | 1.654(3) | 0.3186(6) | 0.053(4) |
| H1A | 0.851135 | 1.651451 | 0.303996 | 0.063 |
| C2 | 0.7469(6) | 1.660(3) | 0.3636(6) | 0.043(4) |
| H2 | 0.720507 | 1.664938 | 0.378777 | 0.052 |
| C3 | 0.8350(6) | 1.492(3) | 0.3578(5) | 0.047(4) |
| H3 | 0.869261 | 1.377600 | 0.368983 | 0.056 |
| C4 | 0.7969(5) | 1.495(3) | 0.3803(5) | 0.036(3) |
| C5 | 0.7347(8) | 1.820(3) | 0.3246(6) | 0.054(5) |
| H5 | 0.699912 | 1.930264 | 0.313452 | 0.065 |
| C6 | 0.8097(6) | 1.330(3) | 0.4217(5) | 0.042(4) |
| C7 | 0.7730(8) | 1.820(4) | 0.3013(7) | 0.067(5) |
| H7 | 0.764589 | 1.929568 | 0.274751 | 0.080 |

**Table S5.** Fitting parameters of TRPL carrier decay curves for different perovskite films.

| Sample | *τ*_1_ (ns) | *A*_1_ (%) | *τ*_2_ (ns) | *A*_2_ (%) | *τ*_avg_ (ns) |
| --- | --- | --- | --- | --- | --- |
| Control | 38.95 | 0.35 | 510.08 | 0.65 | 345.18 |
| BZ | 29.25 | 0.22 | 682.47 | 0.78 | 538.76 |
| TFBZ | 15.45 | 0.13 | 980.67 | 0.87 | 855.19 |

**Table S6.** Fitting parameters of TRPL carrier decay curves for different perovskite films coated with spiro-OMeTAD HTL.

| Samples | *τ*_1_ (ns) | *A*_1_ (%) | *τ*_2_ (ns) | *A*_2_ (%) | *τ*_avg_ (ns) |
| --- | --- | --- | --- | --- | --- |
| Control/HTL | 14.86 | 0.29 | 366.49 | 0.71 | 264.52 |
| BZ/HTL | 8.45 | 0.34 | 274.36 | 0.66 | 183.95 |
| TFBZ/HTL | 6.37 | 0.41 | 157.69 | 0.59 | 95.64 |

**Table S7.** Fitting parameters of EIS impedance spectra of control, BZ-based and TFBZ-based devices.

| Devices | *R*_s_ (Ω) | *R*_tr_ (kΩ) | *R*_rec_ (kΩ) |
| --- | --- | --- | --- |
| Control | 49.38 | 4.25 | 28.84 |
| BZ | 43.62 | 4.11 | 22.24 |
| TFBZ | 37.50 | 2.19 | 15.11 |

**Table S8.** *J*-*V* characteristics of champion devices under reverse scan and forward scan, with an active area of 0.06 cm^2^. The device structure is FTO/SnO_2_/PVK/HTL/Au.

| Device | Scan direction | *V*_oc_ (V) | *J*_sc_ (mA cm^-2^) | | *FF* (%) | *PCE* (%) |
| --- | --- | --- | --- | --- | --- | --- |
| Control | Backward | 1.15 | | 25.12 | 82.4 | 23.80 |
|  | Forward | 1.13 | | 24.98 | 79.8 | 22.52 |
| BZ | Backward | 1.17 | | 25.35 | 83.6 | 24.79 |
|  | Forward | 1.15 | | 25.26 | 82.8 | 24.04 |
| TFBZ | Backward | 1.18 | | 25.47 | 85.0 | 25.54 |
|  | Forward | 1.17 | | 25.42 | 83.4 | 24.81 |

# References

[1] R. Zhao, P. Wang, L. Wang, Y. Zhao, C. Ge, L. Sun, L. Xie, Y. Hua, *Adv. Funct. Mater.* **2024**, *34*, 2307559.

[2] P. Caprioglio, M. Stolterfoht, C. M. Wolff, T. Unold, B. Rech, S. Albrecht, D. Neher, *Adv. Energy Mater*. **2019**, *9*, 1901631.

[3] W. D. Cornell, P. Cieplak, C. I. Bayly, I. R. Gould, K. M. Merz, D. M. Ferguson, D. C. Spellmeyer, T. Fox, J. W. Caldwell, P. A. Kollman, *J. Am. Chem. Soc.* **1995**, *117*, 5179.

[4] T. Lu, F. Chen, *J. Comput. Chem.* **2012**, *33*, 580-592.

[5] G. Kresse, D. Joubert, *Phys. Rev. B* **1999**, *59*, 1758-177.

[6] J. P. Perdew, K. Burke, M. Ernzerhof, *Phys. Rev. Lett.* **1996**, *77*, 3865-3868.

[7] S. Grimme, J. Antony, S. Ehrlich, H. Krieg, *J. Chem. Phys.* **2010**, *132*, 154104.

[8] H. J. Monkhorst, J. D. Pack, *Phys. Rev. B,* **1976**, *13* 5188–5192.
